# Aspiration–attainment gaps predict adolescents’ subjective well-being after transition to vocational education and training in Germany

**DOI:** 10.1371/journal.pone.0287064

**Published:** 2023-06-12

**Authors:** Désirée Nießen, Alexandra Wicht, Clemens M. Lechner

**Affiliations:** 1 GESIS – Leibniz Institute for the Social Sciences, Mannheim, Germany; 2 Federal Institute for Vocational Education and Training, Bonn, Germany; 3 University of Siegen, Siegen, Germany; University of Bamberg, GERMANY

## Abstract

An occupational aspiration–attainment gap (AAG) is defined as a discrepancy between the socioeconomic status (SES) of the aspired occupation and the one attained. We investigated how experiencing an occupational AAG after transition to vocational education and training (VET) affects three domains of subjective well-being (SWB) among adolescents in Germany (general life, job, and income satisfaction). Using longitudinal data from the German Socio-Economic Panel (SOEP), we could track respondents’ SWB during the transition to and during VET. Results from latent growth curve models revealed that both under- and overachievement of aspirations (i.e., negative and positive AAGs) reduced initial levels of SWB after VET entry—especially work-related satisfaction (i.e., income and job satisfaction). Individuals with an AAG (both negative and positive) tended to experience a slightly larger increase in SWB during VET than those who met their aspirations. Overall, our results suggest that the decisive factor for adolescents’ SWB is not the SES of the VET position they attain, but rather whether that position is the exact type of position to which they aspired.

## Introduction

The occupational *aspiration–attainment gap* (AAG) refers to the discrepancy between the socio-economic status (SES) of occupational aspirations and attainment. Three possible constellations can be distinguished: (a) falling short of one’s occupational aspirations (i.e., negative AAG or simply underachievement); (b) exactly realizing one’s occupational aspirations (i.e., no AAG or simply perfect match); and (c) surpassing one’s occupational aspirations (i.e., positive AAG or simply overachievement). Experiencing an AAGs is a widespread but unequally distributed phenomenon among adolescents in vocational education and training (VET) in Germany [1]. Previous studies have shown that, when accessing VET, young people have to make several compromises—for example, in terms of qualification level, SES, career opportunities, job stability, and gender typicality [1–3].

But what are the *consequences* of experiencing an AAG? Although several scholars have argued that experiencing an AAG—at an early career stage—likely entails unfavorable long-term consequences for career development [1, 4], only a few studies have investigated the extent to which occupational AAGs have negative consequences for individuals’ subjective well-being (SWB) as well [5, 6]. The consequences of occupational AAGs for SWB are relevant both for individuals and for employers for two reasons: first, because SWB is an important outcome in its own right; and second, because SWB predicts outcomes such as work motivation [7], goal engagement [8], and goal striving [9]. Low SWB is therefore a likely precursor of job dropout or occupational change [10] and other potentially detrimental career outcomes. Medici et al. [11], for example, showed that individuals in Switzerland with higher VET satisfaction in the final phase of VET were more likely to work in their trained occupations, thus underscoring the importance of early formative experiences during VET for later, often costly, undesired job turnover.

In addition to the scant evidence on the link between AAGs and SWB, little is known about the development of SWB in the event of an AAG. Studies have pointed to adaptive processes that occur even in the face of severe negative events and circumstances. For example, a meta-analysis conducted by Luhmann et al. [12] found that a few years after losing their jobs, many people reached pre-unemployment levels of SWB. Similar patterns of adaptation have been found for non-work stressors such as filial bereavement [13]. However, longitudinal studies tracking SWB over time among individuals who have experienced an AAG are absent from the literature.

In the present study, we investigated the consequences of experiencing an AAG for SWB during the course of VET. Our study was guided by two research questions: (a) Does an occupational AAG impair young people’s SWB when starting VET? (b) If so, does the SWB of apprentices decrease, increase, or remain the same over the course of VET as a function of this AAG? To answer these questions, we used representative longitudinal data from Germany tracking SWB among young people in VET for a period of 2 years.

## Theoretical background

### Consequences of occupational AAGs for SWB and for changes in SWB over time

*Social production function theory* [14] views social status as an essential instrumental goal for achieving well-being, but one that is highly dependent on resources and constraints. Occupational aspirations are a key variable that reflects social status goals in light of a person’s opportunities. Many theoretical models deal with the question of how such occupational aspirations develop. One of them is Gottfredson’s [15] *theory of circumscription*, *compromise and self-creation*. According to the theory, the development of occupational aspirations during childhood and adolescence is divided into three phases, in which aspirations become more and more concrete. First, youth develop a “zone of acceptable alternatives” (p. 91) along the core dimensions of social status and gender in the phase of circumscription. In the phase of compromise, they adapt this zone to their perceived opportunity structures, which depend on their interests, learning experiences as well as individual and sociostructural characteristics and resources. In doing so, they finally prefer, in the phase of self-creation, occupational choices that yield the best person–environment fit (see also [16]).

But what happens when the actual occupational attainment does not match occupational aspirations, which are already the result of an adjustment to perceived opportunity structures? There are several relevant theories on the consequences of discrepancies between desired and actual states. They converge in predicting that discrepancies between occupational aspirations and attainment will impair the SWB of young people in VET, whereas realizing or exceeding these aspirations will enhance SWB. The *level of aspiration theory* assumes that an individual’s level of aspirations serves as a reference point for their feelings of success and failure [17]. Consequently, attainment that surpasses aspirations is perceived as success, whereas attainment that does not meet aspirations is perceived as failure. Likewise, *multiple discrepancies theory* asserts that SWB is a function of discrepancies (gaps) between what individuals have (i.e., attainment) and what they expected to have in the past or want for the future (i.e., aspirations) [18]. Relatedly, *self-discrepancy theory* states that negative emotions depend on the type and magnitude of subjectively perceived discrepancies between the actual/own self-state (i.e., attainment, self-concept) and the ideal self-state (i.e., aspirations, wishes), or between the actual/own self-state and the ought self-state (i.e., duties, responsibilities) [19]. It posits that actual–ideal discrepancies signify the *absence* of positive outcomes (i.e., the non-achievement of expectations), and are associated with feelings such as dissatisfaction and disappointment, whereas actual–ought discrepancies signify the *presence* of negative outcomes (i.e., the expectation of punishment), and are associated with feelings such as fear and restlessness.

Although these theories agree that discrepancies are generally detrimental to SWB, they do not explicitly consider changes in SWB over time while experiencing an (enduring) discrepancy of the kind that an occupational AAG represents. Research on SWB shows that individuals tend to return to a relatively stable baseline level of happiness after positive or negative life experiences—a phenomenon captured by the metaphor *hedonic treadmill* coined by Brickman and Campbell [20] and also known as *hedonic adaptation*. Although these processes of adaptation vary among and within individuals [21], it is conceivable that the initially lower level of SWB in young people who have experienced an occupational AAG gradually returns to a baseline level similar to that of their peers who have not experienced an AAG.

### Previous research on the consequences of occupational AAGs for SWB

Little research has addressed the potential impact of occupational AAGs on SWB. In line with the theoretical perspectives discussed above, the few existing studies suggest that experiencing an occupational AAG leads to lower job satisfaction [6], lower SWB [5], lower enjoyment of VET [22], more depressive symptoms [5, 6], and a decreased likelihood that individuals perceive themselves as “very successful” in their work lives [5].

Evidence on the consequences of overachievement (i.e., attainment that exceeds aspirations) is inconclusive. In a study on the consequences of unrealized occupational goals in the transition to adulthood conducted by Hardie [6], overachievement was found to be associated with higher job satisfaction in one sample and with lower job satisfaction in another. A study examining the impact of occupational AAGs on women’s mental health (i.e., depression, psychological well-being, and purpose in life) at midlife [5] found that there was no difference in mental health between those who had surpassed and those who had met their aspirations. Moreover, women who experienced a large positive occupational AAG were *less* likely to perceive themselves as “very successful” in their work lives than those who met their aspirations [5]. In line with Kahneman and Tversky’s classic dictum from prospect theory that “losses loom larger than gains” [23, p. 279], these inconsistent findings suggest that underachievement (i.e., experiencing a negative AAG) is more relevant to SWB than overachievement.

In addition to the aforementioned studies on the consequences of occupational AAGs for SWB, several studies have investigated the consequences of *educational* AAGs (i.e., discrepancies between aspired and attained levels of education). Similar to the occupational AAG studies, Paat [24] found that educational AAGs were linked to higher depressive symptoms among Mexican immigrant youth in the USA. Another study found that university-educated Canadians who worked in jobs for which they were overqualified had a significant risk of decline in self-rated health over a 4-year period [25]. By contrast, among secondary- or less-educated respondents, differences in occupational attainment were unrelated to differences in the risk of decline in self-rated health [25]. In a sample from the USA, Reynolds and Baird [26] initially found that young adults who experienced an educational AAG had a greater risk of experiencing symptoms of depression—but only at the lowest level of aspirations (i.e., among those who failed to achieve a high school diploma). However, this apparent negative effect of an AAG on mental health disappeared when differences in depressive symptoms associated with educational attainment were additionally controlled for [26]. Likewise, a recent study of German adolescents did not find any differences in life satisfaction between those who experienced an educational AAG and those who did not [27].

However, these studies investigated the consequences of an educational AAG and/or used samples from North America, mostly from the USA—sometimes comprising only women [5] or immigrants [24]—collected several decades ago (assessment of aspirations between 1975 and 1990). Moreover, they focused mostly on a different phase of life than our present study: aspirations at age 35 and attainment and dependent variables at age 53 [5]; aspirations at age 14–22 and attainment and dependent variables at age 27–35 [6]. Thus, the findings of these few studies are not readily generalizable to the potential consequences for SWB of an early career AAG in the highly structured German school and VET system. In addition, these studies were mostly cross-sectional and did not investigate how SWB developed over time as a function of an AAG (for exceptions, see [25, 27]). Some of the studies also looked at quite different outcomes (e.g., [mental] health rather than SWB) or used only global measures of SWB. Because people may evaluate different domains of life differently, a comprehensive view of SWB should go beyond a global evaluation [28] and examine individual domains of the construct. Consequently, further research is needed to close this gap.

### Previous evidence on possible confounding variables

The previous studies discussed so far mostly assumed that experiencing an AAG is causally linked to SWB. In testing such a purported causal effect, it is important to consider potential factors that may act as confounders (or “third-variables”). In this section, we present a brief summary of factors that prior research has found to be related to both the AAG and SWB: the Big Five personality traits and sociostructural characteristics (parental SES, migration background, gender).

A recent study of adolescents beginning VET in Germany reported that lower levels of Big Five Emotional Stability, Agreeableness, and Conscientiousness, and higher levels of Big Five Openness were associated with a higher likelihood of experiencing an occupational AAG/a higher risk of a larger AAG [1]. Moreover, across different studies, the Big Five dimensions have consistently been found to be positively related to domains of SWB—for example, satisfaction with VET [29], job satisfaction (for a review, see [30]), and life satisfaction [31]—although not all dimensions were always equally relevant. This suggests that Big Five personality traits might act as confounders in the relation between the AAG and SWB.

Also, individuals with lower parental SES have been found to be more likely to experience an educational [32] or occupational [1] AAG, whereas higher parental SES has been found to be associated with a larger AAG [1] and higher life satisfaction [33]. Having a migration background has been shown to be related to a higher likelihood of experiencing an occupational AAG and a larger occupational AAG [1]. Furthermore, studies have reported that, compared to Whites, Blacks (and Hispanics) tend to report lower job satisfaction [34] and life satisfaction [35].

Previous findings regarding the relationship between gender, the AAG, and SWB have been inconsistent. Most studies have shown that males have a higher likelihood of experiencing an educational [32] or occupational [1] AAG, and that females are more likely to experience larger educational [36] or occupational [1] AAGs. With respect to SWB, a recent cross-nationally comparative study found higher life satisfaction in females in 18 nations [31]. Other studies have reported, for example, higher VET satisfaction in males [29] or higher job satisfaction in females [37]. Despite the inconsistent findings, it is clear that gender may also play a confounding role in the AAG–SWB relation and should be accounted for.

## The present study

Little is known about the impact of experiencing an occupational AAG on adolescents’ SWB. The present study aimed to close this gap by tracing the development of SWB after the transition to VET among young people in Germany. For this purpose, we used representative longitudinal data from Germany on apprentices who had started their first VET position leading to a full vocational qualification. The data allowed us to track the SWB of adolescents over a period of 2 years (i.e., from VET entry until 2 years after VET entry). Below, we briefly describe the German VET system, the three aspects of SWB we investigated as outcomes, and then outline our specific research questions and hypotheses.

### Country context: The German VET system

VET is an integral part of Germany’s highly structured and stratified education system. Students typically enter VET after completing 9th or 10th grade at a vocationally oriented secondary school track, although other pathways are possible. The dual VET system provides initial vocational training before labor market entry [38]. For an average of 3 years, apprentices learn the practical skills of a specific occupation at a company (on-the-job training) and, in parallel, take theoretical occupation-related and general subjects at a part-time vocational school (schooling) [39]. Different VET positions (i.e., low-skilled occupations such as baker, skilled occupations such as mechatronics technician, professional occupations such as bank clerk) require different school-leaving qualifications, which are usually attained after different years of schooling. Because the VET system in Germany is vertically stratified, not all VET positions are equally accessible [38]. As the transition from school to VET and from VET to the labor market is highly standardized, young people who have completed a certain VET program can typically only take up a job in that particular occupational field. Thus, because occupational upward mobility is very rare in Germany, a VET-related AAG may often persist in later work life [38]—unless individuals close it by switching to a different VET position that better matches their prior aspirations. This potential “long arm” of VET for later careers renders the German VET system an interesting case to study the consequences of occupational AAGs for SWB.

### Outcomes: General life, job, and income satisfaction

SWB is a multi-faceted construct that comprises affective (e.g., positive and negative affect experienced in everyday life) and cognitive-evaluative components (e.g., one’s overall general life satisfaction) [40]. In this study, we focused on the cognitive-evaluative component. We chose this focus partly on theoretical grounds and partly because of data availability: The discrepancy theories on which our hypotheses are based mostly—though not exclusively—refer to the cognitive-evaluative component; and the only SWB measures collected annually in the data we used were satisfaction measures (i.e., referred to the cognitive-evaluative component). In the cognitive-evaluative component, one generally distinguishes global satisfaction and satisfaction with specific domains of life. We considered both, and more specifically, three domains of SWB that are particularly relevant with regard to occupational attainment and further career development: general life satisfaction, job satisfaction, and income satisfaction.

*General life satisfaction* refers to the global assessment of the overall quality (i.e., all aspects) of a person’s life [40, 41]. It can be regarded as the cognitive-evaluative component of SWB [40]. Spector defined *job satisfaction* as “the extent to which people like […] or dislike […] their jobs” [42, p. 2], and the way they feel about different aspects of their jobs, for example, their treatment at work or the demands and challenges of the job. Job satisfaction and income are the two main outcome measures of career success (for a meta-analysis, see [43]), and experiencing high job satisfaction is crucial for further career progression [11]. Finally, *income satisfaction* can be understood simply as the degree to which people are satisfied with their personal income. According to the *effort–reward imbalance* (ERI) model [44], an imbalance between high work effort and low rewards (e.g., through income or recognition) is perceived as unfair, and results in negative feelings (e.g., disappointment, dissatisfaction) and poor health in the long term because of continuous strain reactions in the autonomic nervous system [45]. Thus, low income satisfaction can prove problematic for the further life course and for career progression.

Although related, satisfaction with these three domains does not necessarily need to coincide. Recent studies (i.e., published after the year 2000) have reported correlations of similar—and generally only moderate—magnitude between general life satisfaction and job satisfaction (e.g., *r* = .30 [46]; *r* = .40 [47]), and between general life and income satisfaction (e.g., *r* = .35 [48]; *r* = .41 [47]). Reported correlations between job satisfaction and income satisfaction have been generally somewhat higher (e.g., *r* = .52 [47]; *r* = .60 [49]). These findings suggest that when evaluating satisfaction with their lives in general, their jobs, and their income, individuals consider different information. Hence, examining the three domains of SWB separately provides a more nuanced picture of the consequences of an occupational AAG for SWB than considering only one domain or aggregating the three domains.

### Research questions and hypotheses

We aimed to answer the following research questions: (a) Does experiencing an AAG impair initial levels of three domains of SWB (general life satisfaction, job satisfaction, income satisfaction) among young people when starting VET in Germany (i.e., the intercept of SWB)? (b) Does the SWB of apprentices decrease, increase, or remain the same over the course of VET as a function of the AAG (i.e., the slope of SWB)? We traced SWB over time (i.e., when starting VET and after 1 year and 2 years in VET), thereby enabling us to examine whether adolescents adapt to an AAG over time, and whether patterns of adaptation differ across individuals. To answer these questions, we analyzed two binary contrasts: underachievement versus perfect match, and overachievement versus perfect match.

We preregistered our hypotheses on a project web page on the Open Science Framework (OSF) website (see https://doi.org/10.17605/OSF.IO/VZK92). Our hypotheses relating to the initial levels of SWB built on aspiration theory [17], multiple discrepancies theory [18], and self-discrepancy theory [19], which postulate that unrealized expectations are associated with lower SWB:

*Hypothesis 1a*: Underachievement compared to a perfect match between aspirations and attainment predicts lower initial levels of SWB.
*Hypothesis 1b*: Especially a large AAG (discrepancy between attainment and aspirations ≤ |5|) predicts lower initial levels of SWB.

Our hypotheses relating to changes in SWB over time were based on Brickman and Campbell’s [20] concept of the hedonic treadmill—also known as the process of “hedonic adaptation.” We assumed that initially lower levels of SWB in young people who experienced an AAG gradually return to a baseline level similar to that of their peers who did not experience an AAG:

*Hypothesis 2a*: Underachievement compared to a perfect match between aspirations and attainment predicts a larger positive change in SWB over the course of VET.
*Hypothesis 2b*: A large AAG (discrepancy between attainment and aspirations ≤ |5|) predicts a larger positive change in SWB.

In addition to testing our preregistered hypotheses, we conducted exploratory analyses concerning: (a) associations between the intercept (defined as the level of SWB at the initial observation time when starting VET) and the slope of SWB; (b) similarities and differences in the AAG–SWB associations across different indicators of SWB; and (c) the association between a positive AAG (i.e., overachievement) and SWB. Because previous findings regarding overachievement have been inconclusive, we refrained from formulating specific expectations in this regard. In additional analyses, and in an exploratory fashion, we defined the level of SWB reached at the last observation during VET as the intercept in order to find out whether experiencing an AAG is also a direct predictor of SWB in the final phase of VET.

When testing the effects of the AAG on SWB, we controlled for potential confounders of the AAG–SWB interface that temporally precede the measurement of the AAG: the Big Five personality traits, sociostructural characteristics (parental SES, migration background, gender), year of entering VET, and VET entry before first interview. We expected our hypotheses to be confirmed both with and without control variables.

## Material and methods

### Dataset and sample description

We used data from the German Socio-Economic Panel (SOEP [50]; version 35, https://doi.org/10.5684/soep-core.v35). Conducted annually since 1984, SOEP is an ongoing longitudinal survey of a representative sample now comprising almost 77,000 participants aged 16/17 years and older residing in over 40,000 private households in Germany. Because it covers a wide range of topics, such as occupational biographies, employment, earnings, health, and satisfaction indicators, SOEP is ideally suited to analyze consequences of the AAG for SWB.

SOEP data are personal data that are subject to special protection in the European Union. The independent SOEP Survey Committee approves the SOEP questionnaire every year. In addition, SOEP is institutionally reviewed by the German Council for Social and Economic Data. Participation in the SOEP study is voluntary and based on the informed consent of participants in compliance with data protection legislation. Written informed consent to participate in this study is provided by the participants or, if aged under 18, by their legal guardians. To prevent harm to respondents, the data may be used only for the intended purpose (scientific research) and in an anonymized form.

The survey is refreshed annually with new 16/17-year-old adolescents from the surveyed households. At panel entry, these respondents complete the initial youth questionnaire, which is regularly updated and has included all variables relevant for our analyses since 2006, thereby resulting in a gross sample size of *N* = 6,073. Thus, the first observations for respondents in our sample came from 13 different survey years (i.e., from 2006 to the last available survey year at the time the analyses were carried out, namely, 2018), and all other relevant variables and events such as school graduation also took place at different observation times. We combined these different observation times per respondent for our analyses.

The number of available observations also varied among individuals as well as by outcome: For example, information on satisfaction with income was available for 10 survey years for some individuals and for only 2 survey years for others. For this reason, and consistent with our focus on SWB during VET, we considered only those survey years in which respondents were in VET. The observed VET episodes lasted at most 3 years for 95% of all respondents (*M* = 1.40 years [The average observed VET duration did not necessarily correspond to the actual VET duration either because of missing data or because of survey dropout from a certain point in time.], *SD* = 1.26, Min. = 0 years, Max. = 5 years). As stated in the pre-registration on the OSF website, we originally intended to cover the period between entry into VET and a maximum of 3 years after entry into VET. However, because the number of valid cases providing information on SWB 3 years after VET entry was too small (about 50) due either to VET dropout or panel dropout or to missing information, our analyses of SWB could cover only the period between VET entry and a maximum of 2 years after VET entry. The last column in Table 1 shows the number of persons for whom information was available for each outcome and the number of years during VET for which information was available.

**Table 1:** Descriptive statistics of all measures.

Continuous variables	Min.	Max.	*M*	*SD*	Valid *N*	Missings
General life satisfaction						
1 year before VET	1	10	7.62	1.59	1,141	395
When starting VET	0	10	7.73	1.60	1,089	447
After 1 year in VET	0	10	7.58	1.55	815	721
After 2 years in VET	0	10	7.37	1.63	437	1,099
Job satisfaction						
When starting VET	0	10	7.92	1.87	703	833
After 1 year in VET	0	10	7.53	2.04	784	752
After 2 years in VET	0	10	7.27	2.07	423	1,113
Income satisfaction						
When starting VET	0	10	5.98	2.68	772	764
After 1 year in VET	0	10	6.05	2.54	807	729
After 2 years in VET	0	10	5.74	2.46	435	1,101
AAG-related variables						
Aspirations	16	88	43.71	14.66	1,265	271
Attainment	16	60	39.56	10.45	997	539
Aspiration–attainment gap	32	–63	–4.26	12.25	726	810
Big Five^a^						
Extraversion	1.0	7.0	4.90	1.25	1,516	20
Agreeableness	1.0	7.0	5.35	0.93	1,519	17
Conscientiousness	1.0	7.0	4.95	1.10	1,521	15
Emotional Stability	1.0	7.0	4.05	1.14	1,524	12
Openness	1.5	7.0	4.64	1.00	1,506	30
Parental SES	11.74	88.31	42.44	18.63	683	853
Process time in months between						
VET entry and t_0_ for						
General life satisfaction	0	10	5.15	3.01	1,089	447
Job satisfaction	0	10	6.15	2.71	703	833
Income satisfaction	0	10	5.98	2.80	772	764
VET entry and t_1_ for						
General life satisfaction	9	26	17.04	3.41	815	721
Job satisfaction	9	26	17.09	3.41	784	752
Income satisfaction	9	26	17.04	3.41	807	729
VET entry and t_2_ for						
General life satisfaction	18	39	28.79	3.52	437	1,099
Job satisfaction	18	39	28.77	3.50	423	1,113
Income satisfaction	18	39	28.78	3.53	435	1,101
Categorical variables			% (incl. missings)		*N*	Missings
Dummy AAGs (0)			52,7			810
Perfect match (AAG = 0)	-	-	24.8	-	380	-
Underachievement (AAG < 0)	-	-	15.6	-	240	-
Overachievement (AAG > 0)	-	-	6.9	-	106	-
Dummy AAGs (+/–5)			52.7			810
Perfect match (AAG ≤ |5|)	-	-	30.0	-	461	-
Underachievement (AAG < –5)	-	-	13.0	-	199	-
Overachievement (AAG > 5)	-	-	4.3	-	66	-
Migration background						0
No	-	-	81.0	-	1,245	-
Yes	-	-	19.0	-	291	-
Gender						0
Male	-	-	54.8	-	841	-
Female	-	-	45.2	-	695	-
Year of entering VET						0
Pre-economic crisis (2006–2009)	-	-	21.3	-	327	-
Post-economic crisis recovery (2010–2013)	-	-	33.8	-	520	-
Normalcy (2014–2018)	-	-	44.9	-	689	-
VET entry before first interview						0
After first interview	-	-	76.7	-	1,178	-
Before first interview	-	-	23.3	-	358	-

*Note*. AAG = aspiration–attainment gap, ISEI = International Socio-Economic Index of Occupational Status, SES = socioeconomic status, VET = vocational education and training.

^a^ We recoded the negatively keyed items (*y*_recoded_ = 8—*y*_original_) before computing the unweighted mean score of the Big Five dimensions.

Given the specific longitudinal data structure, we drew on data for all variables between 2006 and 2018, depending on the phase that the respondents were in: Information on personality traits, sociostructural characteristics, and occupational aspirations was collected with the initial youth questionnaire when the respondents were aged 16/17 years and most of them were still at school, and hence before entry into VET. We gathered information on the VET position attained and the year of entering VET for the time when respondents entered VET and after school graduation (t_0_). We gathered information on the consequences of the AAG for SWB for the year of VET entry (t_0_) and 1–2 years after VET entry (t_1_–t_2_). We gathered information on general life satisfaction additionally for the year before VET entry (t_–1_). Table 1 displays the descriptive statistics of all measures in the present sample. S1 Appendix displays the correlations between SWB and all other variables.

We excluded (a) respondents whose last observed episode was a school episode—that is, who dropped out of the panel after school graduation—(leading to *N* = 5,496); (b) respondents for whom no information on the time of school graduation was available (leading to *N* = 4,104); (c) respondents who never entered VET (leading to *N* = 1,928); (d) respondents with inconsistent data (e.g., the VET episode began prior to school graduation; leading to *N* = 1,858); (e) respondents who completed VET before the first survey (leading to *N* = 1,804); and (f) respondents for whom neither information on aspirations, attainment, nor on a single dependent variable was available (resulting in a total *N* of 1,536 respondents).

Respondents’ average age at the time of school graduation was 16.97 years (*SD* = 1.43, Min. = 14, Max. = 22). Respondents graduated with different school-leaving certificates: (a) basic school-leaving qualification (29.6%; German: *Hauptschulabschluss*; International Standard Classification of Education [ISCED-97] Level 2B); (b) intermediate school-leaving qualification (46.0%; German: *Mittlere Reife*; ISCED-97 Level 2A); (c) entrance qualification for a university of applied sciences (6.7%; German: *Fachhochschulreife*; ISCED-97 Level 3A); and (d) general higher education entrance qualification (17.73%, German: *Abitur*; ISCED-97 Level 3A; for an overview of the ISCED-97 levels in the German education system, see [51]).

### Measures

#### Main predictor: Aspiration–attainment gap (AAG)

To operationalize the AAG, we calculated the difference between the SES of the occupation in which an apprenticeship was obtained after VET entry and the SES of the occupation aspired to before VET entry. Taking this difference was possible because both the attained and the aspired occupations were coded according to the SES they confer in terms of the ISEI scores (International Socio-Economic Index of Occupational Status [52]) associated with the corresponding occupation. As a metric variable, the AAG can have a negative value (aspirations higher than attainment; AAG < 0; i.e., underachievement), a positive value (aspirations lower than attainment; AAG > 0; i.e., overachievement), or a value of zero (aspirations equal to attainment; AAG = 0). We computed two dummy variables, and used them to compare, first, *underachievement* (1) and, second, *overachievement* (2) with the reference category *perfect match* (0). Besides the dummies with a zero threshold (i.e., underachievement: AAG < 0, overachievement: AAG > 0, perfect match: AAG = 0), we computed two further dummy AAGs with the threshold of |5|—that is, AAG values between –5 and +5 were not assigned to underachievement or overachievement but to the category perfect match. We compared the effects of these two different AAG thresholds on SWB to find out whether only a certain magnitude of AAG affects SWB. (Note that we could not investigate further thresholds, for example, of |10| because the number of valid cases would have become too small.)

In SOEP, occupational aspirations are measured in two steps. At the age of 16/17, the respondents are asked whether they had an occupational aspiration: “Do you already know what occupation you want to take up?” (our literal translation of “Wissen Sie schon, welchen Beruf Sie ergreifen möchten”; for the freer SOEP translation [“Do (you) have a career aspiration?”], see [53, p. 24]). If this question is answered in the affirmative, the aspired occupation is measured with the open question “What kind of occupation is that? Please state as exact[ly] as possible.” [53, p. 24]. Occupational attainment is assessed annually with the open question “What is your current position/occupation? Please state the exact title in German. […] If you are an apprentice or in vocational training, please state the occupation for which you were trained.” [54, p. 22].

The answers are coded into various standard occupation classification schemes. As a typical measure of SES, and consistent with some earlier studies [1], we decided to use ISEI scores. ISEI takes into account the educational level that a specific occupation requires and the associated earnings of that occupation [55], meaning that higher ISEI scores indicate higher earnings and higher levels of skill requirements and demands. The latest ISEI version of 2008 was developed and validated based on the International Social Survey Program (ISSP). For this purpose, data on occupation, education, and personal earnings of 200,000 working people in 42 countries worldwide between 2002 and 2007 were used [56]. Scores range from 11.56 (*low*), representing gardeners, to 88.96 (*high*), representing judges.

Even though the average salary earned after completing VET in the learned occupation is not the same as the training allowance, the same differences are reflected here as well (among other things, by industry, training year and region; e.g., [57]). The average gross monthly earning for, for example, the low-skilled occupation of carpenter (ISEI score: 26.62) is 732 euros during VET (https://www.ausbildung.de/berufe/tischler/gehalt/#tab-bar-anchor) and 2,883 euros after entering working life (https://web.arbeitsagentur.de/entgeltatlas/beruf/4458). Apprentices in the skilled occupation of industrial mechanic (ISEI score: 31.72) make on average 980 euros gross per month (https://www.ausbildung.de/berufe/industriemechaniker/#gehalt) and 3,950 euros after completing their VET (https://web.arbeitsagentur.de/entgeltatlas/beruf/29056). The occupation of insurance broker (ISEI score: 60.29) belongs to the professional occupations, in which one earns an average of 1,200 euros gross per month during VET (https://www.ausbildung.de/berufe/versicherungsmakler/gehalt/#tab-bar-anchor) and 5.342 euros afterwards (https://web.arbeitsagentur.de/entgeltatlas/beruf/7058).

If respondents’ attainment values represented occupations that cannot be attained without tertiary education (ISEI scores above 64), we set the value on this variable to “missing.” We presumed that these improbable attainment values were due to either completion (e.g., inconsistent response behavior) or coding errors. If there was a missing value for attainment when starting a VET position, we chose the attainment value from the year after VET entry and controlled for the duration of the VET episode—that is, we filled attainment values only if we could be certain that respondents had not switched to a different VET position or to employment. We continued this procedure if there were also missing values for the year after entry into VET, and so forth, controlling each time for the duration of the VET episode.

#### Dependent variables

Our focus was on the consequences of the AAG for respondents’ SWB. We considered these consequences for three domains of SWB (general life satisfaction, job satisfaction, and income satisfaction), which although correlated to a certain extent are independent of each other. In SOEP, current general life satisfaction, job satisfaction, and income satisfaction are surveyed annually with the questions “How satisfied are you with your life, all things considered?” [54, p. 73]; “How satisfied are you with your job?” [54, pp. 4–5]; and “How satisfied are you with your personal income?” [54, pp. 4–5] on a response scale ranging from 0 (*completely dissatisfied*) to 10 (*completely satisfied*). As the S1 Appendix shows, although the three satisfaction domains are correlated to some extent (.30 ≤ *r ≤* .36) at the same observation time after VET entry, they are not interchangeable measures.

We used information on the satisfaction variables from the year that respondents entered VET until up to 2 years after VET entry. When doing so, we controlled for the duration of the VET episode, and we used information on, for example, job satisfaction after 2 years in VET only if we could be certain that respondents had not switched to a different VET position or to employment when job satisfaction was assessed. It is noteworthy that there were a substantial number of missing values especially for the job satisfaction and income satisfaction variables at the time when starting a VET position (see Table 1). This was due either to the fact that the respondents did not provide information on satisfaction (i.e., item missingness) or that respondents’ VET episode began in the year of panel entry, be it before the survey or after the survey (so that the question about job satisfaction and income satisfaction was not asked). Job satisfaction and income satisfaction are not surveyed in the initial youth questionnaire [53] but rather in the subsequent questionnaire administered to each eligible member of the household, which respondents receive for the first time 1 year after panel entry [54].

#### Time-invariant control variables

We included the measures described below in the analyses as potential confounding variables for the AAG and its consequences. All these variables were measured prior to entry into VET.

*Big Five personality traits*. Since 2006, the 15-item Big Five Inventory-SOEP (BFI-S) [58] and one additional item for Openness have been used to measure the Big Five personality traits in all 16/17-year-olds when they complete the youth questionnaire for the first time. The dimensions Extraversion, Agreeableness, Conscientiousness, and Emotional Stability are assessed with three items each, whereas Openness is assessed with four items in order to capture this dimension more broadly. The items are answered using a 7-point rating scale that ranges from 1 (*strongly disagree*) to 7 (*strongly agree*). BFI-S has satisfactory psychometric properties [58]. In the present study, the internal consistencies of the dimensions ranged between Cronbach’s alpha = .45 (Agreeableness) and .72 (Extraversion), which can be deemed sufficient for short scales [59]). We used each respondent’s first available Big Five scores before they entered VET.

*Sociostructural characteristics*. To assess respondents’ sociostructural characteristics, we used parental SES, migration background, and gender.

In SOEP, *parental SES* is assessed with the open question “What [is your father’s/mother’s position/occupation or what] was your father’s/mother’s last occupation?” [53, p. 36]. The answers are coded into standard occupational classification schemes, such as the International Standard Classification of Occupations (ISCO) [60], ISEI [61], and the Standard International Occupational Prestige Scale (SIOPS) [52, 62]. To keep the classification between parental SES and occupational aspirations equivalent, we used ISEI scores. In cases where the mother’s and father’s ISEI scores differed, we used the higher score. We coded *migration background* as 0 (*no*) versus 1 (*yes*) based on information about the respondent’s country of birth and citizenship. The *gender* of the respondent was coded as 0 (*male*) or 1 (*female*).

*Year of VET entry*. The year of entering VET varied among respondents and ranged between 2006 and 2018. This variable was included in the analyses to compensate for seasonal effects (e.g., of the economy or the labor market). For this purpose, we combined between 4 and 5 sequential years in order to represent three different phases: 2006–2009 (pre-economic crisis), 2010–2013 (post-economic crisis recovery), and 2014–2018 (normalcy). We tested two binary contrasts: *2014–2018 normalcy* (0; reference category) versus *2010–2013 post-economic crisis recovery* (1), and *2014–2018 normalcy* (0) versus *2006–2009 pre-economic crisis* (1).

*VET entry before first interview*. We included the binary variable VET entry before first interview in the analyses to compensate for potential causal effects because some respondents had already started a VET position before their first interview—that is, before all other independent variables had been collected. We coded the variable as 0 (*no*) versus 1 (*yes*).

#### Time-varying control variable process time

We included the process time in months between VET entry and t_0_, between VET entry and t_1_, and between VET entry and t_2_ in the analyses because the time that elapsed between the single observations differed among respondents.

### Analyses

#### Main analyses

We used conditional latent growth curve (LGC) modeling and Mplus version 8.4 [63] to analyze the relationships between the AAG, the control variables, and both the initial level of SWB and changes in SWB during VET. LGC modeling allowed us to analyze the systematic intra-individual changes in repeated measures over time, on the one hand, and inter-individual differences in these changes, on the other. LGC models predict the random intercept (i.e., the initial level) and the random linear slope (i.e., mean growth rate of change) by means of other (time-invariant and/or time-varying) covariates (conditional LGCs). In this way, we can explain in more detail why some individuals experience a certain type of change in SWB and why others do not. We defined the initial observation time when starting VET (t_0_) as the intercept. This allowed us to predict the initial level of SWB (i.e., the intercept) after entry into VET as well as the change in (i.e., the slope of) SWB during the course of VET over a period of 2 years (t_0_–t_2_; i.e., when starting VET to 1–2 years in VET) over these initial levels.

**Fig 1:**
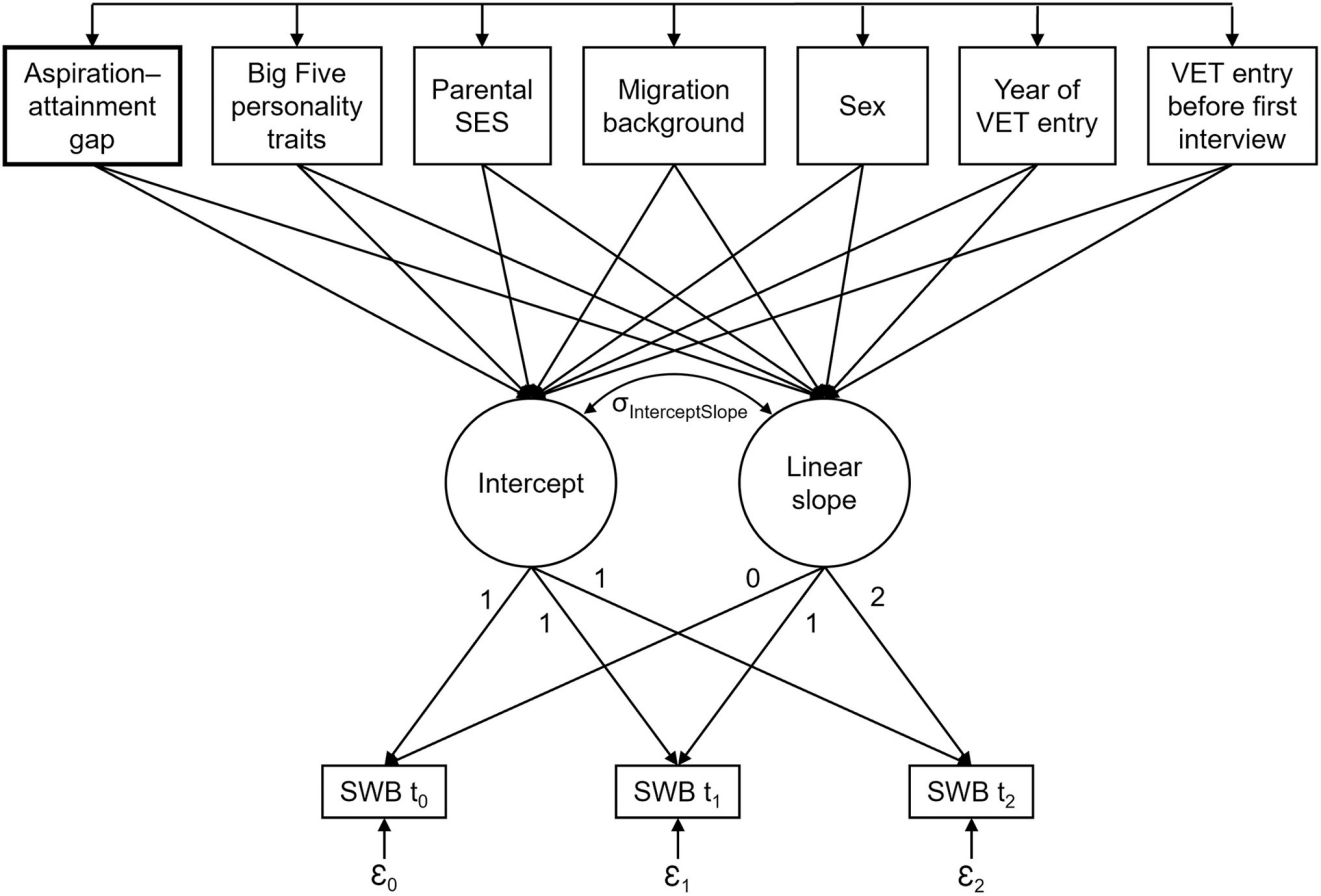
Schematic depiction of the third latent growth curve model with time-invariant predictors (Model III). *Note*. VET = vocational education and training, SWB = subjective well-being, σ_InterceptSlope_ = intercept–slope covariance, t = measurement time, Ɛ_t_ = residual variance.

For each dependent variable (i.e., general life satisfaction, job satisfaction, and income satisfaction), we ran the following analyses: In the first step, we modeled an unconditional LGC model without predictors (Model I) to test how well a LGC model fit the data. In the second step, we used the two AAG dummy variables as manifest time-invariant predictors of SWB (Model II) to examine whether there was an effect of AAG on SWB, thereby using the “auxiliary” option to identify the missing data correlates of aspirations and attainment in addition to the analysis variables (see [63]). In the third step, we conducted further analyses that additionally included the control variables as manifest time-invariant predictors (Model III) to investigate whether these variables changed the effect of AAG on SWB. To predict general life satisfaction only, in the fourth step, we included pre-VET general life satisfaction at t_–1_ as additional manifest predictor (Model IV) to control for unobserved heterogeneity. The model estimates are reported in the S2 Appendix. A schematic depiction of the most complex model with predictor variables is displayed in Fig 1. The model formula is as follows:

$${\text{SWB}}_{\text{t}}=\text{Intercept}+\lambda_{\text{t}}\text{Slope}+\left(\text{Predictor}\;\text{variables}\;+\right)\;\varepsilon_{\text{t}}$$

SWB_t_ is the observed SWB score at time t, λ_t_ is the elapsed time in years, and ε_t_ is the residual variance. The intercept is composed of Intercept = μ_α_ + ζ_α_, and the slope is composed of Slope = μ_β_ + ζ_β_, where μ is the mean, ζ the variance, α the model-implied latent intercept factor, and β the latent linear slope factor.

We computed Models II–IV twice: first, with the two dummy AAG variables with the threshold of zero with underachievement as AAG < 0, overachievement as AAG > 0, and perfect match as AAG = 0. Second, we used the two dummy AAG variables with the threshold of |5| with underachievement as AAG < –5, overachievement as AAG > 5, and perfect match as –5 ≤ AAG ≤ 5. We compared the effects of these two different AAG thresholds on SWB to find out whether a large AAG (≥ |5|) affected SWB more than the more conservative computation of an AAG (< 0).

We modeled the LGCs in a linear fashion with fixed time scores (i.e., loadings of the slopes) of 0 (t_0_), 1 (t_1_), and 2 (t_2_). The loadings of the intercepts of the three observation times of each SWB domain were 1. We used maximum likelihood estimation with robust standard errors (MLR) and restricted all latent variances (i.e., of the intercept and slope) to positive values to facilitate model convergence. The variances of the independent variables were modeled as free parameters to be estimated using default starting values. Furthermore, we used full information maximum likelihood (FIML; e.g., [64]) estimation in our analyses to handle missing values on single items of the independent as well as the dependent variables (unit nonresponse and item nonresponse). FIML is a method that estimates only the population parameters of interest that would most likely yield the estimated values from the sample data analyzed (e.g., variance, covariance). This estimation is performed by maximizing the likelihood function for the observed data and based on both the available complete data and the implied values of the missing data [65]. Prior to the analyses, we recoded negatively keyed items, such as reverse-keyed Big Five items.

The following points differed from the pre-registration: (a) Instead of focusing on the period between VET entry and a maximum of 3 years after VET entry, we focused on the period between VET entry and a maximum of 2 years after VET entry because valid cases for SWB values 3 years after VET entry were too small (about 50). (b) Instead of using the difference score on the metric AAG variable, we used two sets of dummy AAG variables with different AAG thresholds (0 and |5|) and compared the effects of these two thresholds on SWB because the sample sizes and variances within the group of AAG < 0 and AAG > 0 were too small. (c) We did not center the continuous independent variables. (d) We did not control for cognitive ability, because cognitive ability has not been shown to be a confounding variable of the AAG in previous research [1].

#### Sensitivity analyses

**Fig 2:**
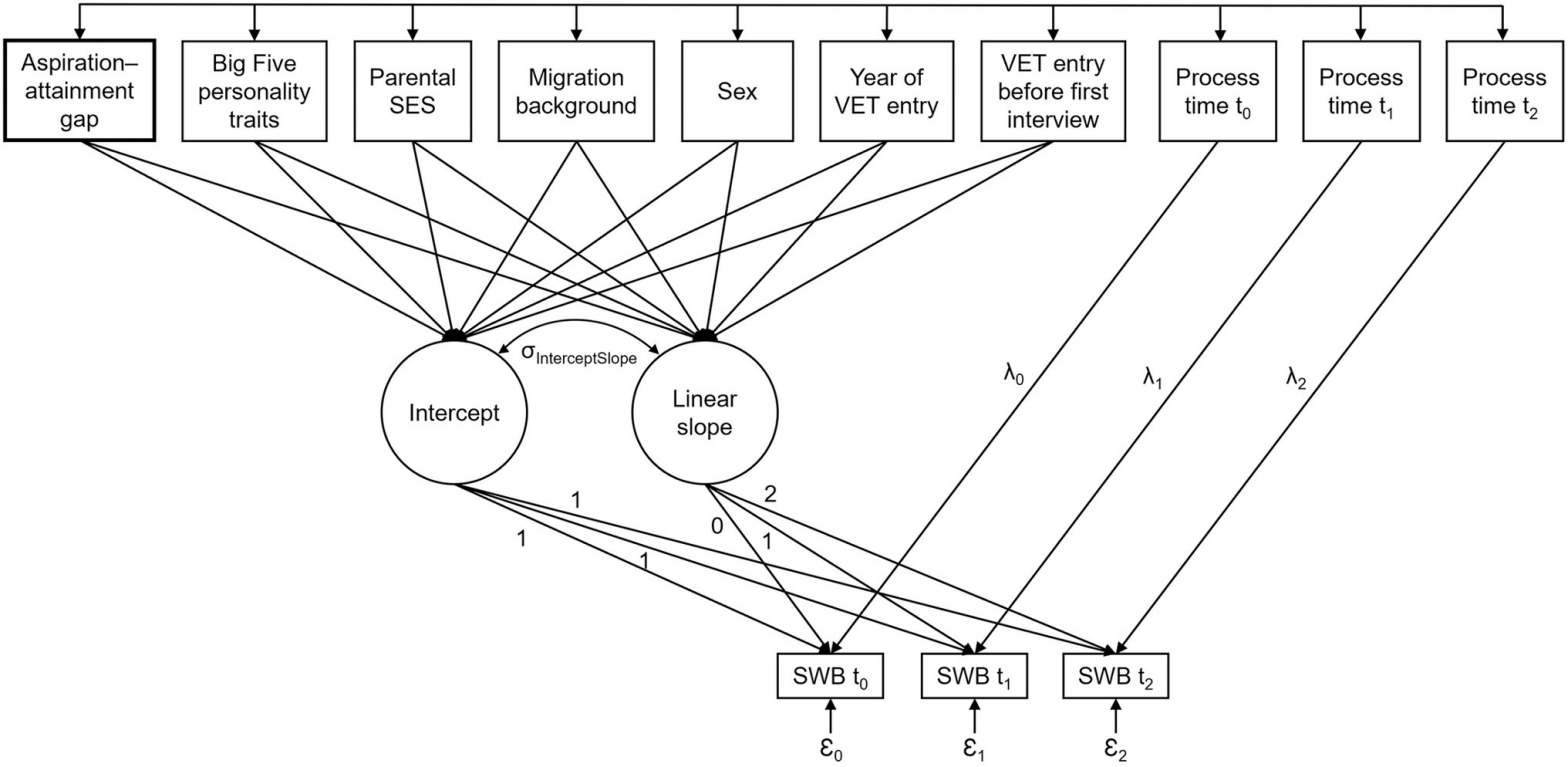
Schematic depiction of the third latent growth curve model with time-invariant and time-varying predictors (Model III). *Note*. VET = vocational education and training, SWB = subjective well-being, σ_InterceptSlope_ = intercept–slope covariance, t = measurement time, λ_t_ = loading, Ɛ_t_ = residual variance.

Because the elapsed (i.e., process) time between the single observations differed among respondents, we computed all models (i.e., Models I–III and I–IV, respectively) a second time as robustness checks, while including the process time variables as manifest time-varying predictors of SWB, thereby assigning the elapsed time to each measurement occasion of SWB as depicted in Fig 2. The results of these sensitivity analyses fully confirmed those of the main analyses reported below and are available as Mplus outputs in the S3 Appendix.

## Results

### Prevalence of the AAG

**Fig 3:**
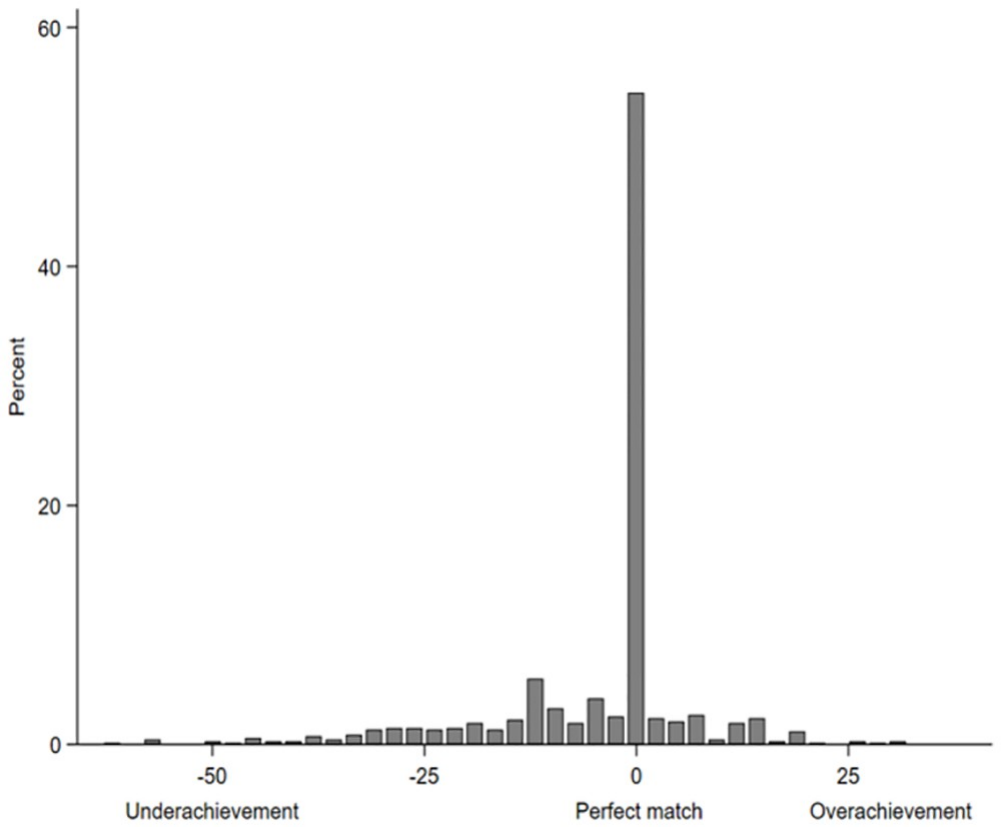
Percentages of the distribution of the aspiration–attainment gap. *Note*. As a metric variable, the peak in the middle includes both the zeros and a few additional observations with values close to zero. *N* = 726.

As Fig 3 shows, for the proportion of the sample for which both an aspiration and an attainment value were available (about 50%), the ISEI score of the attained VET position perfectly matched the ISEI score of their aspired occupation in 52.3% cases—that is, these respondents fully realized their aspirations in terms of SES. In 14.6% of cases, respondents even surpassed their aspirations by attaining VET positions with ISEI scores that were up to 32 points higher than that of their aspired occupations (i.e., overachievement). By contrast, 33.1% of respondents fell short of their aspirations and attained VET positions with ISEI scores that were up to 63 points lower than that of their aspired occupations (i.e., underachievement). The percentages of the distribution of the components of the AAG (i.e., aspirations and attainment) can be found in the S4 Appendix.

### Predicting the initial level of and change in SWB

We started by regressing the initial level (i.e., intercept) and the mean growth rates of change (i.e., linear slope) of three domains of SWB (general life satisfaction, job satisfaction, and income satisfaction) on the predictor variables. S5 Appendix shows the fit indices of the models (Models I–III).

#### Unconditional model (Model I)

The first model we analyzed was the unconditional LGC model without predictors (Model I). The model results are displayed in the S6 Appendix. According to the benchmarks proposed by Hu and Bentler [66], this basis model showed a very good model fit for all dependent variables (see the S5 Appendix). Although the intercept–slope covariance was significant only for income satisfaction, job satisfaction and general life satisfaction tended in the same—namely, negative—direction. This negative intercept–slope covariance in connection with a negative slope in all SWB domains showed that higher initial levels of SWB were related to up to 1.2 scale points steeper declines, or more decline over time, and lower initial levels of SWB were related to slower declines, or a less steep negative slope.

#### The AAG predicting SWB (Model II)

The second model we analyzed was the conditional LGC model with the two dummy AAGs as predictors (Model II). The model results are displayed in the S7 Appendix. As shown in that table, both underachievement and overachievement were statistically significant predictors of lower levels of general life satisfaction and job satisfaction after VET entry (i.e., the intercept) in the case of the AAG threshold of zero, thus confirming Hypothesis 1a, which states that underachievement compared to perfect match predicts lower initial levels of SWB. This finding means that a negative and a positive AAG (i.e., underachievement and overachievement of occupational aspirations in terms of SES) were associated with 0.3–0.5 lower initial levels of general life satisfaction and 0.3–0.7 lower initial levels of job satisfaction. In the case of the AAG threshold of |5|, the effect of underachievement remained about the same, whereas the effect of overachievement decreased slightly and was no longer significant. However, overachievement still showed larger negative effects on the initial level of SWB than underachievement—namely, −0.4 scale points for general life satisfaction and −0.6 scale points for job satisfaction.

Income satisfaction showed a different picture: The negative significant effect of underachievement on the intercept in the case of the zero threshold even increased in the case of the |5|-threshold, thus confirming Hypothesis 1b and indicating that falling short of occupational aspirations by at least 5 ISEI score points (i.e., a large AAG) was associated with 0.9 lower scale points in income satisfaction. Furthermore, the non-significant tendency of overachievement on the intercept in the case of the zero threshold increased substantially in the case of the |5|-threshold, with 0.8 scale points lower income satisfaction for those who exceeded their aspirations by at least 5 ISEI score points. This indicates that the effect on income satisfaction became stronger when AAG values deviated more (by at least 5 ISEI score points) from zero (i.e., became larger). Overall, the influence of the AAG on SWB was strongest for income satisfaction, followed by job satisfaction, and was weakest for general life satisfaction.

Only job satisfaction showed, at threshold |5|, a marginally significant positive effect of overachievement on the linear slope (i.e., mean growth rate of change), indicating that surpassing occupational aspirations by at least 5 ISEI score points led to a 0.4 scale points higher increase in job satisfaction over time. Thus, Hypotheses 2a and 2b could not be confirmed, as the AAG did not predict the development of SWB over time.

The intercept–slope covariance again showed the strongest negative effect for income satisfaction, but this effect was slightly decreased compared to Model I. Higher initial levels of SWB were related to 1.1 scale points steeper declines in income satisfaction over time.

#### The AAG and the covariates predicting SWB (Model III)

**Fig 4:**
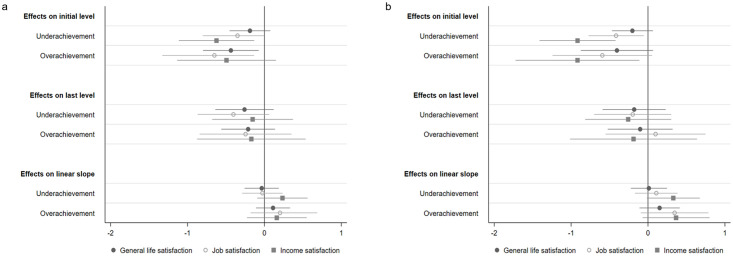
Three domains of SWB regressed on the AAG (Model III)—t0 as intercept and t2 as intercept. *Note*. Unstandardized regression coefficients with 95% confidence interval. *N* = 1,536. A: AAG-threshold 0. B: AAG-threshold |5|.

The third model we analyzed was the conditional LGC model with the two dummy AAGs and all control variables as predictors (Model III). The model results are displayed in the S8 Appendix. The regression coefficients of under- and overachievement on the intercept and the linear slope of SWB are additionally depicted in Fig 4. When additionally taking the covariates into account, the picture was similar to that in Model II in terms of the directions and strength of the effects, thus confirming Hypothesis 1a that underachievement (and also overachievement) predicts lower initial levels of SWB after VET entry. Again, the impact of the AAG on income satisfaction was strongest, followed by job satisfaction, and the impact of the AAG on general life satisfaction was weakest.

Taking a look at the three SWB domains separately revealed, first, that the negative effects of under- and overachievement on the initial level of general life satisfaction decreased slightly compared to Modell II, and that there was a significant influence of overachievement only on the intercept at threshold 0. However, the effect at threshold |5| was still recognizable and was comparable to that in Model II. Surpassing occupational aspirations led to 0.4 scale points lower general life satisfaction. Second, the negative effects of under- and overachievement on the initial level of job satisfaction were very similar to those in Model II, and increased only very slightly compared to that model. Falling short of or surpassing occupational aspirations led to 0.4–0.7 scale points lower job satisfaction. Third, the negative effects of under- and overachievement on the initial level of income satisfaction even increased substantially—especially at threshold |5|—thereby revealing that falling short of or exceeding aspirations by at least 5 ISEI score points (i.e., a large AAG) was related to almost 1 scale point lower income satisfaction. Hypothesis 1b could be confirmed especially in the case of income satisfaction and job satisfaction, as large AAGs were associated with lower initial levels of SWB.

Similar to Model II, there was no significant impact of the AAG on the mean rate of change in SWB for any of the three SWB domains. However, there were nevertheless small positive tendencies that even increased at threshold |5|: Exceeding aspirations was related to 0.2–0.4 scale points higher increases in SWB over time, and falling short of aspirations was related to up to 0.3 scale points higher increases in SWB over time (lowest for general life satisfaction and highest for income satisfaction). Thus, Hypotheses 2a and 2b could not be confirmed, as SWB increased only slightly over the course of VET as a function of the AAG (and of large AAGs).

The intercept–slope covariance was significant for income satisfaction (threshold 0 and |5|) and for job satisfaction (threshold 0 only but there was also a tendency for threshold |5| in the same—namely negative—direction). In contrast to Models I and II, the linear slope was positive in both SWB domains (but for job satisfaction at threshold |5|), implying that higher initial levels of SWB were related to 0.6–1.1 scale points lower increase, or slower rates of positive change, in SWB over time, and lower initial levels of SWB were related to steeper increases or a steeper positive slope.

### Predicting the last level of SWB

Second, we predicted the level of general life satisfaction, job satisfaction, and income satisfaction reached at the last observation at t_2_ to investigate whether experiencing an AAG predicted the level of SWB in the final phase of VET, and whether there were differences compared to the models where the initial level of SWB (i.e., t_0_) was defined as the intercept. In the following, we describe only the most important results that differ compared to the models with the intercept defined as t_0_, and only for Model III including the covariates. The model coefficients that differ compared to the model predicting the initial level of SWB are displayed in the S6 Appendix (for Model I), in the S7 Appendix (for Model II), and in the S8 Appendix (for Model III). The regression coefficients of under- and overachievement on the intercept of SWB (Model III) are additionally depicted in Fig 4.

In almost all cases, the effects of under- and overachievement on SWB were discernibly lower than for the models where the initial level was the intercept. However, with one exception, the direction of the effects remained the same (0.1 to −0.4 scale points vs. −0.2 to −0.9 scale points). Thus, apprentices who experienced an AAG were no longer less satisfied than their peers who had obtained the VET positions in the occupations to which they aspired, although a slight tendency toward lower SWB was still apparent.

## Discussion

The present study examined the consequences of an occupational AAG (i.e., the discrepancy between the SES of the aspired and attained occupation) for three SWB domains (general life satisfaction, job satisfaction, and income satisfaction) among apprentices in Germany. We analyzed the relevance of underachievement (i.e., negative AAG) and overachievement (i.e., positive AAG) compared to achieving one’s aspirations (i.e., experiencing no AAG) on both the initial and the final level of SWB after VET entry as well as on the development of SWB over the course of VET for a period of up to 2 years.

### How many youth experience an AAG?

Before discussing the predictive power of the AAG for SWB, it should first be emphasized that the distribution of the AAG we found is almost identical to that found with findings from a recent study [1] using another representative data set from Germany. What makes the finding even more remarkable is that this study [1] only had graduates from intermediate secondary schools (*Realschule*) from a single cohort in their sample, whereas ours included all school types (general secondary school [*Hauptschule*], intermediate secondary school [*Realschule*], academically oriented secondary schools [*Gymnasium*]) and school leavers from 13 different survey years. This suggests that the AAG is a replicable phenomenon in Germany, similarly distributed across all school types and over time.

### Experiencing an AAG decreases apprentices’ initial and final levels of SWB

Results fully supported our preregistered Hypotheses 1a and 1b relating to the initial levels of SWB, which were based on aspiration theory [17], multiple discrepancy theory [18], and self-discrepancy theory [19]. Consistent with evidence from the literature [5, 6], unmet expectations⸺both underachievement and, notably, also overachievement⸺were associated with lower initial levels of SWB during the first year of VET. We found the strongest negative effects of an AAG on SWB for income satisfaction, followed by job satisfaction—that is, work-related SWB—particularly for large negative AAGs. For general life satisfaction, we found only small effects.

It may be surprising at first glance that we also observed a clear negative impact of *over*achievement on all three SWB domains. This finding is at odds with the level of aspiration theory [17], according to which realized or exceeded aspirations are perceived as success and therefore enhance SWB. A possible explanation for this is that the SES of the VET position attained is not decisive for apprentices’ SWB; rather, the decisive factor is whether or not apprentices obtain the exact occupation (i.e., VET position) to which they aspired, regardless of whether the position they attained confers a higher (or lower) social status than the occupation to which they originally aspired. A previous study with an adult female sample reported similar results—namely, that both under- and overachievement were related to lower perceptions of being very successful in work life [5]. This raises the question of what actually determines whether a negative or positive AAG will negatively impact SWB. We can speculate that the decisive factor is whether the attained occupation matches the one to which an individual aspired in terms of the occupational field in question (see also [67]) with the specific work tasks it entails and in terms of the skills, interests, and other characteristics required for the occupation. If, for example, someone wants to become a baker but then trains to become a bank clerk, their SES is much higher (overachievement), but instead of standing in a fragrant bakery and kneading dough, they have to spend their working hours at the counter or in the office. The higher status does not make the person happy because they wanted to do something else. This assumption is in line with previous findings showing that AAGs with respect to the occupational field are most relevant for both enjoyment of and dropout from VET [22, 68].

Moreover, the results indicate that overachievement has an even stronger negative effect on job satisfaction and general life satisfaction than underachievement does. At threshold of |5| ISEI points, overachievement and underachievement have an equally strong effect on income satisfaction that was even stronger than their effect at threshold 0. Thus, our results do not support the assumption that underachievement is more detrimental to SWB than overachievement. One possible reason for this finding is that overachievers might perceive the tasks associated with their VET positions to be too challenging and demanding, which is reflected in their job satisfaction and general life satisfaction. The same applies to income satisfaction. Income may not be evaluated in absolute terms but rather against the background of the individual’s own effort.

Because both under- and overachievement showed similar patterns, and because not achieving the desired VET position—rather than the status difference between the occupation aspired to and the occupation in which a VET position was attained—appeared to be crucial, we ran Model III including the confounding variables again (intercept defined as initial level of SWB only), combining the groups of under- and overachievers and testing them against those with a perfect match (no AAG). As expected, the effect sizes lay roughly in the middle between the effects of under- and overachievement and were all negative (general life satisfaction: −0.3, job satisfaction: −0.5, income satisfaction: −0.6 to −0.9 scale points; see the S8 Appendix). However, the effects of the combined group were more often statistically significant—inter alia, the impact of the AAG on the change in income satisfaction was also significant (−0.3 scale points)—presumably because the sample sizes were larger. These results reinforce the interpretation that neither the direction of the AAG nor the extent of the AAG in terms of SES is the key determinant of whether it will negatively impact SWB, but whether or not apprentices are able to obtain the exact type of position to which they aspired.

### Experiencing an AAG is largely unrelated to the development of SWB over the course of VET

The results did not confirm our preregistered Hypotheses 2a and 2b relating to changes in SWB over time, which were based on the concept of the hedonic treadmill [20]. Although for all three satisfaction measures there was a tendency for the initially lower SWB levels of individuals who experienced an AAG to gradually return to the levels of their peers who did not experience an AAG, few of the associations between the AAG and the slope of SWB were statistically significant. Nonetheless, the general pattern is in line with the observation that SWB often returns to a stable baseline level after positive or negative life experiences such as filial bereavement or unemployment [12, 13], a phenomenon also known as a process of hedonic adaptation.

Although the effect sizes were rather small for the association between AAG and SWB development, in most cases there were somewhat higher tendencies toward dissatisfaction in the case of large AAGs and of overachievement. In all, however, both overachievers and underachievers were more dissatisfied at the beginning of VET, as reflected in the clear and significant differences (compared with those who experienced a perfect match between aspirations and attainment). At the end of VET, those with an AAG remained slightly more dissatisfied, but these differences were no longer significant and decreased substantially, as indicated by the smaller effect sizes of the AAG at the last SWB level compared with the initial level. In this context, it should be noted that the remaining sample size for the last observation had decreased considerably. This finding also corresponds to our above interpretation that not realizing occupational aspirations has negative consequences on SWB, regardless of the SES of the occupations in question, and that this is related to occupational field, interests, and skills.

### Implications

To sum up, the present investigation reveals that both negative and positive occupational AAGs (i.e., under- and overachievement) impair the level of SWB after entry into VET and—to a lesser degree—in the final phase of VET. They also predict a tendency toward a larger positive change in SWB over the course of VET. By looking at satisfaction with three domains, we were able to provide a nuanced picture of the consequences for SWB of an occupational AAG among adolescents in an early-career stage over a period of 2 years in Germany. This constitutes a substantial contribution to the literature on educational transitions as does the finding that not only under- but also overachievement was equally detrimental to SWB. A theoretical implication is that there is an urgent need for more advanced multidimensional theoretical models of transition success that take into account not only the objective side (e.g., SES, prestige, earnings) but also the subjective side (e.g., the AAG, interests, suitability). In addition, theoretical models should establish a stronger link between different types of AAGs (e.g., social status, gender type, field of work) and SWB. In doing so, future studies and future theory development should also develop a more nuanced view of the importance of different domains of satisfaction, taking into account aspiration theories, given that work-related satisfaction is more strongly influenced by the AAG than, for example, general life satisfaction.

Because the AAG is a highly prevalent phenomenon [1] that affects SWB, which in turn is a predictor of various work-related outcomes such as work motivation [7] and later undesired job turnover [11], and because VET determines career options within a specific occupational field, it is likely that SWB serves as a mediator between the AAG and long-term consequences for career progression. Hence, to better understand this phenomenon, it is a task of future research to scrutinize this mediating role of the AAG, for example, in dropout from VET (see [22]), completion of VET, and other “harder” career outcomes. The findings of this research will be relevant for policymakers and employers who wish to initiate interventions to improve SWB during VET to support further career development—especially because the wishes and expectations of school leavers do not necessarily coincide with the VET positions available on the labor market (i.e., employment realities) [69].

### Limitations of the present study and directions for future research

The present study has some limitations. First, the focus was on the level and development of SWB after the transition from school to VET and during VET, and for this purpose we used data from school leavers whose school-leaving qualifications ranged from the basic school-leaving qualification (*Hauptschulabschluss*) to the general higher education entrance qualification (*Abitur*). As the latter qualification entitles school leavers to attend university, future research is needed to ascertain whether our findings can be generalized to other educational transitions and replicated in other national contexts.

Second, although we analyzed three different aspects of SWB, these aspects all represented the cognitive-evaluative dimension of SWB. For reasons of data availability, we could not include measures of affective SWB, such as positive and negative affect. Future studies should gain additional insights by comparing potential effects of the AAG on affective SWB to those we observed on cognitive-evaluative SWB.

Furthermore, our results suggest that the AAG no longer has a (large) impact on the level of SWB at the end of VET or on the development of SWB during VET. However, the statistical power of our analyses was somewhat limited by missing data arising inter alia from panel attrition. These missing data also meant that we were unable to observe the development of SWB during VET over a longer (ideally 3-year) period as originally intended. Thus, future research should explore this tendency further over a longer period. Indeed, the literature emphasizes that underachievers often downgrade their aspirations over time to better cope with the disappointments they experience [4]. Whether this is also the case for overachievers in the opposite way remains to be clarified.

## Conclusion

The results of the present study reveal differences in apprentices’ level of SWB (most notably for work-related satisfaction) as a function of an occupational AAG at the beginning of VET: Those who did not obtain a VET position in their aspired occupation were more dissatisfied at the beginning of VET than those who did. At the end of VET, there were no longer any significant differences between the two groups, but a slight tendency in the same direction was observable. Particularly strong discrepancies between aspirations and attainment led to higher dissatisfaction. However, it was not a particular type of AAG (overachievement vs. underachievement) that appeared to impair SWB, but rather the fact that a VET position in the aspired occupation was not obtained, regardless of the SES that that position conferred. Thus, status-related success does not seem to be as important (or crucial) for young people regarding the consequences for their SWB. It might rather be the fulfilment in terms of occupational interests and personal goals, which is most relevant to the magnitude of SWB. Related to this finding, we found no clear evidence of different changes in SWB between individuals with and without an AAG, but again there was a slight tendency for an increase in SWB over the course of VET for those with an AAG. Thus, our results show that experiencing an AAG—whether overachievement or underachievement—is detrimental to SWB, and tends to remain so (although with waning effect sizes) over a period of 2 years. Further research should extend these findings by examining both the impact of an occupational AAG and the mediating role of SWB on subsequent career development.

## Supporting information

S1 Appendix Correlations between three domains of subjective well-being and all other variables included in the study. (PDF)

S2 Appendix Unstandardized coefficients of the latent growth curve models for general life satisfaction regressed on the aspiration–attainment gap, the covariates, and pre-VET general life satisfaction (Model IV). (PDF)

S3 Appendix Mplus outputs of the sensitivity analyses of the third latent growth curve models with time-varying control variables (Models I–IV). (TXT)

S4 Appendix Percentages of the distribution of aspirations and attainment. (PDF)

S5 Appendix Fit indices of the latent growth curve Models I–III. (PDF)

S6 Appendix Unstandardized coefficients of the unconditional latent growth curve models (Model I). (PDF)

S7 Appendix Unstandardized coefficients of the latent growth curve models for three domains of subjective well-being regressed on the aspiration–attainment gap (Model II). (PDF)

S8 Appendix Unstandardized coefficients of the latent growth curve models for three domains of subjective well-being regressed on the aspiration–attainment gap and covariates (Model III). (PDF)
