# Supplementary material for: Aspiration–attainment gaps predict adolescents’ subjective well-being after transition to vocational education and training in Germany
Source: PLoS One. 2023 Jun 12;18(6):e0287064. doi: 10.1371/journal.pone.0287064 (PMC10259778; doi:10.1371/journal.pone.0287064)
Supplement: S1 Appendix — (PDF) [file pone.0287064.s001.pdf]

S1 Appendix

Correlations Between Three Domains of Subjective Well-Being and all Other Variables Included in the Study

	r [95% CI] (pairwise *N*)									
	General life satisfaction				Job satisfaction			Income satisfaction		
	t_{-1}	t_0	t_1	t_2	t_0	t_1	t_2	t_0	t_1	t_2
General life satisfaction t_0	.35 [.29, .41] (773)									
General life satisfaction t_1	.28 [.21, .36] (575)	.42 [.36, .48] (699)								
General life satisfaction t_2	.29 [.18, .39] (307)	.34 [.24, .42] (364)	.49 [.41, .55] (429)							
Job satisfaction t_0	.16 [.09, .23] (689)	.30 [.23, .37] (696)	.24 [.15, .33] (458)	.17 [.05, .30] (237)						
Job satisfaction t_1	.12 [.04, .20] (556)	.25 [.18, .32] (669)	.33 [.26, .39] (780)	.27 [.18, .36] (418)	.46 [.39, .53] (449)					
Job satisfaction t_2	.11 [–.01, .22] (296)	.31 [.21, .40] (350)	.21 [.11, .30] (415)	.39 [.30, .47] (420)	.37 [.26, .48] (228)	.49 [.41, .56] (407)				
Income satisfaction t_0	.23 [.16, .30] (756)	.30 [.24, .36] (764)	.24 [.15, .32] (488)	.06 [–.06, .18] (255)	.36 [.29, .42] (697)	.14 [.06, .23] (470)	.07 [–.05, .20] (245)			
Income satisfaction t_1	.17 [.09, .24] (570)	.18 [.10, .25] (688)	.33 [.27, .39] (803)	.18 [.09, .27] (425)	.22 [.13, .30] (453)	.33 [.27, .39] (781)	.12 [.02, .21] (411)	.57 [.51, .63] (483)		
Income satisfaction t_2	.17 [.06, .28] (306)	.23 [.13, .32] (361)	.25 [.16, .34] (427)	.28 [.18, .36] (432)	.17 [.04, .29] (235)	.30 [.22, .39] (416)	.28 [.19, .37] (422)	.45 [.35, .55] (253)	.59 [.52, .65] (422)	

(continued)

	r [95% CI] (pairwise *N*)									
	General life satisfaction				Job satisfaction			Income satisfaction		
	t_{-1}	t_0	t_1	t_2	t_0	t_1	t_2	t_0	t_1	t_2
Aspirations	.00<br>[–.06, .07]<br>(895)	–.01<br>[–.08, .06]<br>(858)	–.01<br>[–.09, .07]<br>(615)	.01<br>[–.10, .12]<br>(328)	–.01<br>[–.10, .08]<br>(502)	–.04<br>[–.13, .04]<br>(586)	.02<br>[–.09, .14]<br>(315)	.04<br>[–.04, .13]<br>(558)	.06<br>[–.02, .14]<br>(606)	.01<br>[–.09, .12]<br>(325)
Attainment	–.00<br>[–.07, .07]<br>(783)	–.02<br>[–.09, .05]<br>(880)	.06<br>[–.01, .13]<br>(759)	.08<br>[–.02, .17]<br>(425)	–.02<br>[–.09, .06]<br>(659)	–.01<br>[–.08, .06]<br>(741)	.04<br>[–.06, .14]<br>(416)	.09<br>[.02, .16]<br>(699)	.09<br>[.02, .16]<br>(753)	.09<br>[–.01, .18]<br>(425)
Aspiration–attainment gap	.02<br>[–.07, .10]<br>(537)	.01<br>[–.07, .08]<br>(649)	.02<br>[–.06, .10]<br>(559)	.01<br>[–.10, .12]<br>(316)	.02<br>[–.08, .11]<br>(458)	.04<br>[–.05, .12]<br>(543)	.02<br>[–.09, .13]<br>(308)	.09<br>[.00, .18]<br>(485)	.04<br>[–.05, .12]<br>(552)	.07<br>[–.05, .17]<br>(315)
Underachievement (threshold 0)	–.06<br>[–.14, .03]<br>(537)	–.04<br>[–.11, .04]<br>(649)	–.07<br>[–.15, .02]<br>(559)	–.05<br>[–.16, .06]<br>(316)	–.05<br>[–.14, .05]<br>(458)	–.12<br>[–.20, –.03]<br>(543)	–.06<br>[–.17, .06]<br>(308)	–.11<br>[–.20, –.02]<br>(485)	–.10<br>[–.18, –.02]<br>(552)	–.09<br>[–.20, .02]<br>(315)
Overachievement (threshold 0)	–.10<br>[–.18, –.02]<br>(537)	–.07<br>[–.15, .00]<br>(649)	–.06<br>[–.14, .03]<br>(559)	.04<br>[–.07, .15]<br>(316)	–.12<br>[.21, –.03]<br>(458)	–.01<br>[–.09, .08]<br>(543)	–.02<br>[–.13, .10]<br>(308)	.01<br>[–.08, .10]<br>(485)	–.02<br>[–.10, .07]<br>(552)	–.01<br>[–.12, .10]<br>(315)
Underachievement (threshold |5|)	–.08<br>[–.16, .00]<br>(537)	–.05<br>[–.13, .02]<br>(649)	–.05<br>[–.13, .03]<br>(559)	–.04<br>[–.15, .07]<br>(316)	–.07<br>[–.16, .03]<br>(458)	–.12<br>[–.20, –.03]<br>(543)	–.03<br>[–.14, .08]<br>(308)	–.14<br>[–.23, –.05]<br>(485)	–.12<br>[–.20, –.03]<br>(552)	–.11<br>[–.22, .00]<br>(315)
Overachievement (threshold |5|)	–.06<br>[–.15, .02]<br>(537)	–.05<br>[–.13, .03]<br>(649)	–.05<br>[–.13, .04]<br>(559)	.07<br>[–.05, .18]<br>(316)	–.09<br>[–.18, .00]<br>(458)	.02<br>[–.07, .10]<br>(543)	.05<br>[–.06, .16]<br>(308)	–.03<br>[–.12, .06]<br>(485)	–.03<br>[–.11, .06]<br>(552)	.04<br>[–.08, .15]<br>(315)
Extraversion	.09<br>[.03, .15]<br>(1,132)	.09<br>[.03, .15]<br>(1,078)	.09<br>[.02, .15]<br>(804)	.10<br>[.00, .19]<br>(429)	.10<br>[.03, .18]<br>(695)	.02<br>[–.05, .09]<br>(772)	.05<br>[–.05, .15]<br>(415)	–.01<br>[–.08, .06]<br>(763)	–.03<br>[–.10, .04]<br>(795)	–.05<br>[–.14, .05]<br>(427)
Agreeableness	.13<br>[.07, .19]<br>(1,133)	.16<br>[.10, .22]<br>(1,080)	.12<br>[.05, .18]<br>(805)	.08<br>[–.02, .17]<br>(429)	.08<br>[.00, .15]<br>(696)	.09<br>[.02, .16]<br>(774)	.14<br>[.04, .23]<br>(415)	.13<br>[.06, .20]<br>(764)	.11<br>[.04, .18]<br>(797)	.10<br>[.01, .19]<br>(427)
Conscientiousness	.16<br>[.10, .21]<br>(1,135)	.13<br>[.07, .19]<br>(1,082)	.09<br>[.02, .16]<br>(807)	.05<br>[–.05, .14]<br>(430)	.08<br>[.01, .15]<br>(697)	.09<br>[.02, .16]<br>(775)	.12<br>[.02, .21]<br>(416)	.05<br>[–.02, .12]<br>(765)	.08<br>[.01, .15]<br>(798)	.07<br>[–.02, .17]<br>(428)

(continued)

	r [95% CI] (pairwise *N*)									
	General life satisfaction				Job satisfaction			Income satisfaction		
	t_{-1}	t_0	t_1	t_2	t_0	t_1	t_2	t_0	t_1	t_2
Emotional Stability	.14 [.09, .20] (1,136)	.14 [.08, .19] (1,084)	.16 [.09, .22] (808)	.20 [.11, .29] (431)	.11 [.04, .19] (698)	.07 [–.00, .14] (776)	.13 [.03, .22] (417)	.04 [–.03, .12] (766)	.04 [–.04, .10] (799)	.07 [–.02, .17] (429)
Openness	.08 [.02, .14] (1,121)	.08 [.02, .14] (1,076)	.02 [–.05, .09] (801)	.02 [–.08, .11] (429)	.07 [–.01, .14] (692)	.02 [–.05, .09] (769)	–.03 [–.13, .07] (415)	.06 [–.02, .13] (760)	.03 [–.04, .10] (792)	.01 [–.09, .10] (427)
Parental SES	.03 [–.06, .12] (464)	.02 [–.07, .11] (443)	.01 [–.10, .12] (304)	–.03 [–.20, .13] (140)	–.04 [–.17, .08] (246)	–.14 [–.25, –.03] (291)	–.23 [–.38, –.06] (136)	.15 [.03, .27] (269)	.17 [.06, .28] (297)	.23 [.06, .38] (140)
Migration background	.10 [.04, .16] (1,141)	.05 [–.01, .11] (1,089)	.07 [00., .14] (815)	.01 [–.09, .10] (437)	.06 [–.02, .13] (703)	–.05 [–.12, .02] (784)	.04 [–.05, .14] (423)	.01 [–.07, .08] (772)	.01 [–.06, .08] (807)	–.00 [–.09, .09] (435)
Female	–.08 [–.13, –.02] (1,141)	–.05 [–.11, .01] (1,089)	–.03 [–.10, .04] (815)	–.08 [–.17, .01] (437)	–.05 [–.13, .02] (703)	–.08 [–.15, –.01] (784)	–.04 [–.14, .06] (423)	–.02 [–.09, .05] (772)	–.02 [–.09, .05] (807)	–.06 [–.15, .04] (435)
VET entry 2006–2009 (Pre-economic crisis)	–.07 [–.13, –.01] (1,141)	–.06 [–.12, –.00] (1,089)	–.09 [–.16, –.02] (815)	–.06 [–.15, .03] (437)	–.05 [–.12, .03] (703)	–.03 [–.10, .04] (784)	–.07 [–.16, .03] (423)	–.09 [–.16, –.02] (772)	–.09 [–.15, –.02] (807)	–.12 [–.21, –.03] (435)
VET entry 2010–2013 (Post-economic crisis recovery)	.05 [–.01, .11] (1,141)	.00 [–.06, .06] (1,089)	.03 [–.04, .10] (815)	–.00 [–.10, .09] (437)	.00 [–.07, .08] (703)	.02 [–.05, .09] (784)	.02 [–.08, .11] (423)	.04 [–.03, .11] (772)	.01 [–.06, .08] (807)	.04 [–.06, .13] (435)
VET entry before first interview	–.04 [–.10, .01] (1,141)	.13 [.07, .18] (1,089)	.06 [–.01, .13] (815)	.00 [–.09, .10] (437)	–.01 [–.08, .07] (703)	–.03 [–.10, .04] (784)	–.02 [–.11, .08] (423)	–.01 [–.08, .06] (772)	.07 [–.00, .14] (807)	.06 [–.03, .16] (435)

Note. SES = socioeconomic status, VET = vocational education and training. *N* = 1,536.
