# Supplementary material for: Aspiration–attainment gaps predict adolescents’ subjective well-being after transition to vocational education and training in Germany
Source: PLoS One. 2023 Jun 12;18(6):e0287064. doi: 10.1371/journal.pone.0287064 (PMC10259778; doi:10.1371/journal.pone.0287064)
Supplement: S2 Appendix — (PDF) [file pone.0287064.s002.pdf]

S2 Appendix

Unstandardized Coefficients of the Latent Growth Curve Models for General Life Satisfaction Regressed on the Aspiration–Attainment Gap, the Covariates, and Pre-VET General Life Satisfaction (Model IV)

	General life satisfaction					
	Threshold 0			Threshold +/−5		
	Coef.	*SE*	*p*	Coef.	*SE*	*p*
Intercept (t_0) on						
Underachievement	−0.105	0.128	.414	−0.112	0.129	.386
Overachievement	−0.253	0.188	.178	−0.248	0.243	.309
Intercept (t_2) on						
Underachievement	−0.165	0.183	.369	−0.084	0.198	.672
Overachievement	−0.096	0.182	.600	−0.013	0.216	.953
Intercept (t_0) on						
Conscientiousness	0.052	0.040	.196	0.054	0.040	.171
Extraversion	0.059	0.041	.147	0.060	0.041	.141
Agreeableness	**0.168**	0.052	.001	**0.166**	0.052	.001
Openness	0.053	0.047	.265	0.050	0.047	.286
Emotional Stability	**0.114**	0.045	.012	**0.118**	0.046	.010
Parental SES	0.002	0.004	.627	0.002	0.004	.620
Migration background	0.158	0.123	.198	0.152	0.122	.212
Female	0.006	0.095	.952	0.015	0.095	.872
Post-economic crisis recovery	−0.147	0.108	.173	−0.148	0.108	.170
Pre-economic crisis	**−0.258**	0.115	.025	**−0.255**	0.114	.025
VET entry before 1st interview	**0.531**	0.175	.002	**0.540**	0.171	.002
General life satisfaction t_{-1}	**0.299**	0.040	<.001	**0.301**	0.040	<.001
Intercept (t_2) on						
Conscientiousness	−0.040	0.055	.462	−0.038	0.055	.482
Extraversion	0.056	0.058	.328	0.055	0.058	.339
Agreeableness	0.058	0.074	.431	0.057	0.074	.441
Openness	−0.033	0.067	.621	−0.040	0.067	.557
Emotional Stability	**0.263**	0.066	<.001	**0.262**	0.067	<.001
Parental SES	−0.006	0.006	.337	−0.005	0.006	.406
Migration background	0.069	0.170	.686	0.081	0.172	.636
Female	0.029	0.135	.828	0.033	0.136	.807
Post-economic crisis recovery	**−0.378**	0.167	.024	**−0.363**	0.166	.029
Pre-economic crisis	**−0.476**	0.164	.004	**−0.469**	0.163	.004
VET entry before 1st interview	0.020	0.182	.915	0.051	0.184	.784
General life satisfaction t_{-1}	**0.233**	0.057	<.001	**0.239**	0.056	<.001

(continued)

	General life satisfaction					
	Threshold 0			Threshold +/−5		
	Coef.	*SE*	*p*	Coef.	*SE*	*p*
Linear slope on						
Underachievement	−0.030	0.111	.788	0.014	0.116	.905
Overachievement	0.079	0.115	.494	0.118	0.135	.383
Linear slope on						
Conscientiousness	−0.046	0.032	.155	−0.046	0.032	.149
Extraversion	−0.001	0.033	.964	−0.002	0.033	.943
Agreeableness	−0.055	0.043	.202	−0.055	0.043	.202
Openness	−0.043	0.040	.284	−0.045	0.039	.255
Emotional Stability	0.075	0.039	.058	0.072	0.040	.067
Parental SES	−0.004	0.003	.281	−0.003	0.003	.326
Migration background	−0.045	0.105	.670	−0.035	0.105	.736
Female	0.012	0.080	.882	0.009	0.080	.911
Post-economic crisis recovery	−0.115	0.097	.235	−0.108	0.097	.266
Pre-economic crisis	−0.109	0.099	.271	−0.107	0.098	.276
VET entry before 1st interview	**−0.256**	0.100	.010	**−0.245**	0.100	.015
General life satisfaction t_{-1}	−0.033	0.035	.342	−0.031	0.034	.368
Intercept–slope						
Covariance (t_0)	−0.178	0.125	.154	**−0.175**	0.068	.010
Covariance (t_2)	0.306	0.158	.053	0.303	0.157	.054
Means						
Underachievement	0.347	0.016	<.001	0.289	0.015	<.001
Overachievement	0.152	0.013	<.001	0.096	0.011	<.001
Conscientiousness	4.950	0.028	<.001	4.950	0.028	<.001
Extraversion	4.899	0.032	<.001	4.900	0.032	<.001
Agreeableness	5.351	0.024	<.001	5.351	0.024	<.001
Openness	4.638	0.026	<.001	4.638	0.026	<.001
Emotional Stability	4.047	0.029	<.001	4.047	0.029	<.001
Parental SES	43.160	1.105	<.001	42.953	1.120	<.001
Migration background	0.189	0.010	<.001	0.189	0.010	<.001
Female	0.452	0.013	<.001	0.452	0.013	<.001
Post-economic crisis recovery	0.339	0.012	<.001	0.339	0.012	<.001
Pre-economic crisis	0.213	0.010	<.001	0.213	0.010	<.001
VET entry before 1st interview	0.233	0.011	<.001	0.233	0.011	<.001
General life satisfaction t_{-1}	7.507	0.116	<.001	7.503	0.114	<.001
Intercepts						
Intercept (t_0)	3.267	0.483	<.001	3.218	0.475	<.001
Intercept (t_2)	4.876	0.687	<.001	4.779	0.676	<.001
Linear slope	0.805	0.416	.053	0.780	0.409	.056

(continued)

	General life satisfaction					
	Threshold 0			Threshold +/−5		
	Coef.	*SE*	*p*	Coef.	*SE*	*p*
Variances						
Underachievement	0.224	0.006	<.001	0.201	0.007	<.001
Overachievement	0.125	0.009	<.001	0.083	0.009	<.001
Conscientiousness	1.215	0.042	<.001	1.215	0.042	<.001
Extraversion	1.552	0.052	<.001	1.552	0.052	<.001
Agreeableness	0.856	0.032	<.001	0.856	0.032	<.001
Openness	0.993	0.033	<.001	0.993	0.033	<.001
Emotional Stability	1.309	0.044	<.001	1.309	0.044	<.001
Parental SES	347.554	14.902	<.001	347.111	14.519	<.001
Migration background	0.154	0.006	<.001	0.154	0.006	<.001
Female	0.248	0.001	<.001	0.248	0.001	<.001
Post-economic crisis recovery	0.224	0.004	<.001	0.224	0.004	<.001
Pre-economic crisis	0.168	0.006	<.001	0.168	0.006	<.001
VET entry before 1st interview	0.179	0.006	<.001	0.179	0.006	<.001
General life satisfaction t_{-1}	2.546	0.160	<.001	2.549	0.160	<.001
Residual variances						
General life satisfaction t_0	1.165	0.213	<.001	1.169	0.163	<.001
General life satisfaction t_1	1.270	0.135	<.001	1.275	0.137	<.001
General life satisfaction t_2	1.112	0.258	<.001	1.113	0.223	<.001
Intercept (t_0)	0.968	0.211	<.001	0.964	0.134	<.001
Intercept (t_2)	1.224	0.277	<.001	1.220	0.276	<.001
Linear slope	0.242	0.116	.038	0.239	0.053	<.001

Note. VET = vocational education and training. *N* = 1,536. Regression coefficients and intercept–slope covariances significant at the $p < .05$ level are in bold type. Fit indices of the model with aspiration–attainment gap (AAG) threshold of 0: $\chi^2(15) = 4.617$, $p = .995$, comparative fit index (CFI) = 1.000, root-mean-square error of approximation (RMSEA) = .000, standardized root-mean-square residual (SRMR) = .005, Bayesian information criterion (BIC) = 68,819.882. Fit indices of the model with AAG threshold of |5|: $\chi^2(15) = 5.227$, $p = .990$, CFI = 1.000, RMSEA = .000, SRMR = .005, BIC = 68,270.625.
