# Supplementary material for: Aspiration–attainment gaps predict adolescents’ subjective well-being after transition to vocational education and training in Germany
Source: PLoS One. 2023 Jun 12;18(6):e0287064. doi: 10.1371/journal.pone.0287064 (PMC10259778; doi:10.1371/journal.pone.0287064)
Supplement: S4 Appendix — (PDF) [file pone.0287064.s004.pdf]

S4 Appendix

Percentages of the Distribution of Aspirations and Attainment

A

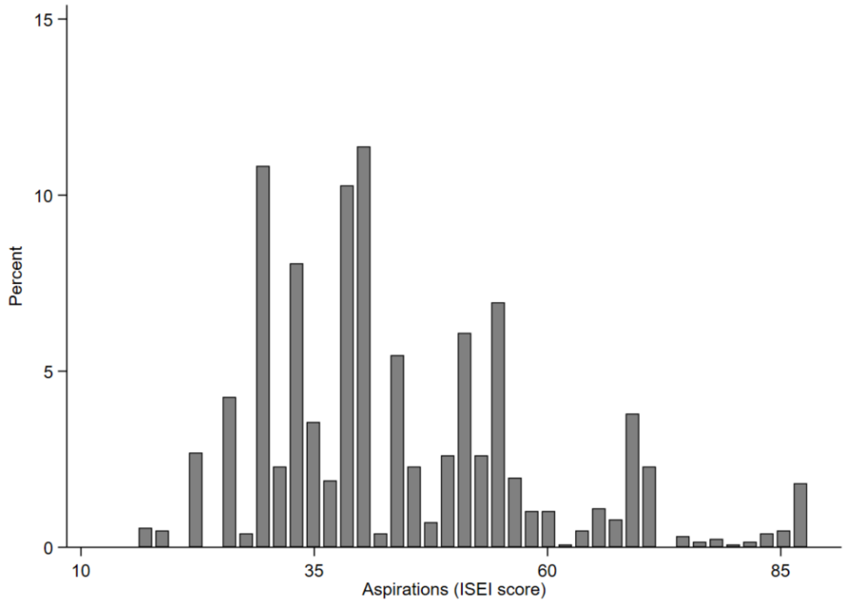

B

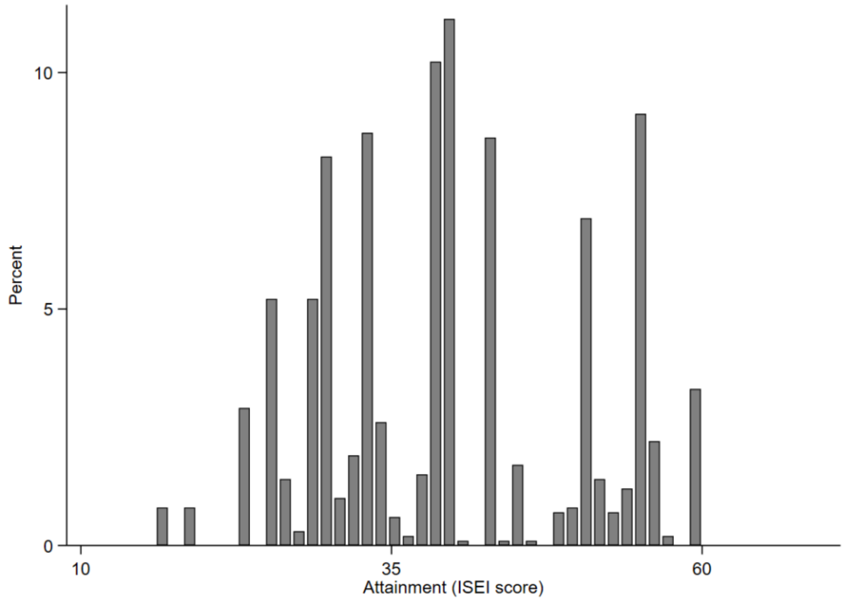

Note. ISEI = International Socio-Economic Index of Occupational Status. A: Distribution of aspirations (*N* = 1,265). B: Distribution of attainment (*N* = 997).
