# Supplementary material for: Aspiration–attainment gaps predict adolescents’ subjective well-being after transition to vocational education and training in Germany
Source: PLoS One. 2023 Jun 12;18(6):e0287064. doi: 10.1371/journal.pone.0287064 (PMC10259778; doi:10.1371/journal.pone.0287064)
Supplement: S5 Appendix — (PDF) [file pone.0287064.s005.pdf]

S5 Appendix

Fit Indices of the Latent Growth Curve Models I–III

	df	χ^2	*p*	CFI	RMSEA	SRMR	BIC
Model I							
General life satisfaction	1	0.004	.948	1.000	.000	.001	8,592.899
Job satisfaction	1	1.397	.237	.997	.019	.012	7,864.152
Income satisfaction	1	0.323	.570	1.000	.000	.005	9,208.292
Model II							
General life satisfaction							
AAG-threshold 0	3	1.311	.727	1.000	.000	.008	27,123.527
AAG-threshold +/−5	3	2.086	.555	1.000	.000	.010	26.568,184
Job satisfaction							
AAG-threshold 0	3	5.099	.165	.992	.021	.021	26,395.080
AAG-threshold +/−5	3	5.384	.146	.989	.023	.023	25,837.174
Income satisfaction							
AAG-threshold 0	3	1.031	.794	1.000	.000	.006	27,730.384
AAG-threshold +/−5	3	1.187	.756	1.000	.000	.006	27,162.963
Model III							
General life satisfaction							
AAG-threshold 0	14	4.485	.992	1.000	.000	.005	64,039.694
AAG-threshold +/−5	14	5.370	.980	1.000	.000	.006	63,039.694
Job satisfaction							
AAG-threshold 0	14	21.242	.096	.994	.018	.015	63,929.135
AAG-threshold +/−5	14	19.564	.145	.995	.016	.015	63,377.329
Income satisfaction							
AAG-threshold 0	14	6.310	.958	1.000	.000	.014	65,265.732
AAG-threshold +/−5	14	6.252	.960	1.000	.000	.014	64,702.880

Note. Model I = unconditional latent growth curve (LGC), Model II = conditional LGC model with the aspiration–attainment gap (AAG) as predictor, Model III = conditional LGC model with the AAG and control variables as predictors. CFI = comparative fix index, RMSEA = root-mean-square error of approximation, SRMR = standardized root-mean-square residual, BIC = Bayesian information criterion.
