# Supplementary material for: Aspiration–attainment gaps predict adolescents’ subjective well-being after transition to vocational education and training in Germany
Source: PLoS One. 2023 Jun 12;18(6):e0287064. doi: 10.1371/journal.pone.0287064 (PMC10259778; doi:10.1371/journal.pone.0287064)
Supplement: S6 Appendix — (PDF) [file pone.0287064.s006.pdf]

S6 Appendix

Unstandardized Coefficients of the Unconditional Latent Growth Curve Models (Model I)

	General life satisfaction			Job satisfaction			Income satisfaction		
	Coef.	*SE*	*p*	Coef.	*SE*	*p*	Coef.	*SE*	*p*
Intercept–slope									
Covariance (t_0)	−0.254	0.147	.083	−0.496	0.289	.087	**−1.171**	0.360	.001
Covariance (t_2)	−0.334	0.181	.066	0.573	0.293	.051	−0.562	0.360	.119
Means									
Intercept (t_0)	7.724	0.047	<.001	7.916	0.066	<.001	6.108	0.088	<.001
Intercept (t_2)	7.377	0.066	<.001	7.198	0.088	<.001	5.661	0.100	<.001
Linear slope	−0.173	0.038	<.001	−0.359	0.053	<.001	−0.224	0.059	<.001
Variances									
Intercept (t_0)	1.376	0.230	<.001	2.455	0.469	<.001	5.594	0.569	<.001
Intercept (t_2)	1.535	0.321	<.001	2.609	0.470	<.001	4.376	0.575	<.001
Linear slope	0.294	0.135	.030	0.535	0.248	.031	0.867	0.303	.004
Residual variances									
Satisfaction t_0	1.178	0.233	<.001	1.081	0.453	.017	1.768	0.525	.001
Satisfaction t_1	1.277	0.142	<.001	2.244	0.250	<.001	2.663	0.241	<.001
Satisfaction t_2	1.088	0.269	<.001	1.660	0.453	<.001	1.703	0.532	.001

Note. N_{life} = 1,209; N_{job} = 1,047; N_{income}= 1,101. Intercept–slope covariances significant at the $p < .05$ level are in bold type.
