# Supplementary material for: Aspiration–attainment gaps predict adolescents’ subjective well-being after transition to vocational education and training in Germany
Source: PLoS One. 2023 Jun 12;18(6):e0287064. doi: 10.1371/journal.pone.0287064 (PMC10259778; doi:10.1371/journal.pone.0287064)
Supplement: S7 Appendix — (PDF) [file pone.0287064.s007.pdf]

S7 Appendix

Unstandardized Coefficients of the Latent Growth Curve Models for Three Domains of Subjective Well-Being Regressed on the Aspiration–Attainment Gap (Model II)

	General life satisfaction						Job satisfaction						Income satisfaction					
	Threshold 0			Threshold +/−5			Threshold 0			Threshold +/−5			Threshold 0			Threshold +/−5		
	Coef.	*SE*	*p*	Coef.	*SE*	*p*	Coef.	*SE*	*p*	Coef.	*SE*	*p*	Coef.	*SE*	*p*	Coef.	*SE*	*p*
Intercept (t_0) on																		
Underachievement	**−0.274**	0.130	.035	**−0.269**	0.133	.043	**−0.336**	0.167	.044	**−0.369**	0.175	.035	**−0.612**	0.239	.010	**−0.864**	0.244	<.001
Overachievement	**−0.474**	0.190	.013	−0.400	0.249	.109	**−0.651**	0.264	.014	−0.570	0.338	.092	−0.439	0.323	.173	−0.792	0.408	.052
Intercept (t_2) on																		
Underachievement	−0.207	0.187	.267	−0.101	0.200	.614	−0.420	0.223	.060	−0.247	0.242	.307	−0.353	0.260	.174	−0.421	0.273	.123
Overachievement	−0.092	0.187	.622	0.071	0.219	.746	−0.112	0.299	.709	0.272	0.316	.390	−0.240	0.344	.486	−0.146	0.398	.713
Linear slope on																		
Underachievement	0.033	0.107	.757	0.084	0.113	.458	−0.042	0.127	.741	0.061	0.135	.650	0.130	0.159	.417	0.221	0.168	.187
Overachievement	0.191	0.116	.099	0.235	0.138	.088	0.270	0.185	.145	0.421	0.215	.050	0.100	0.193	.605	0.323	0.215	.133
Intercept–slope																		
Covariance (t_0)	−0.241	0.143	.093	−0.234	0.146	.108	−0.477	0.293	.103	−0.436	0.298	.144	**−1.125**	0.364	.002	**−1.066**	0.424	.012
Covariance (t_2)	0.336	0.180	.061	0.330	0.179	.066	0.559	0.292	.055	0.528	0.296	.075	0.549	0.363	.130	0.560	0.364	.124
Means																		
Underachievement	0.333	0.016	<.001	0.276	0.015	<.001	0.333	0.016	<.001	0.276	0.015	<.001	0.337	0.016	<.001	0.282	0.015	<.001
Overachievement	0.149	0.013	<.001	0.094	0.011	<.001	0.149	0.013	<.001	0.094	0.011	<.001	0.150	0.013	<.001	0.095	0.011	<.001
Intercepts																		
Intercept (t_0)	7.884	0.070	<.001	7.834	0.063	<.001	8.132	0.099	<.001	8.078	0.087	<.001	6.392	0.144	<.001	6.438	0.122	<.001
Intercept (t_2)	7.455	0.092	<.001	7.398	0.083	<.001	7.336	0.125	<.001	7.229	0.114	<.001	5.810	0.145	<.001	5.786	0.131	<.001
Linear slope	−0.214	0.055	<.001	−0.218	0.049	<.001	−0.398	0.079	<.001	−0.424	0.070	<.001	−0.291	0.093	.002	−0.326	0.081	<.001

(continued)

	General life satisfaction						Job satisfaction						Income satisfaction					
	Threshold 0			Threshold +/−5			Threshold 0			Threshold +/−5			Threshold 0			Threshold +/−5		
	Coef.	*SE*	*p*	Coef.	*SE*	*p*	Coef.	*SE*	*p*	Coef.	*SE*	*p*	Coef.	*SE*	*p*	Coef.	*SE*	*p*
Variances																		
Underachievement	0.224	0.006	<.001	0.201	0.007	<.001	0.223	0.006	<.001	0.201	0.007	<.001	0.223	0.006	<.001	0.200	0.007	<.001
Overachievement	0.125	0.009	<.001	0.083	0.009	<.001	0.125	0.009	<.001	0.083	0.009	<.001	0.125	0.009	<.001	0.083	0.009	<.001
Residual variances																		
Satisfaction t_0	1.192	0.232	<.001	1.201	0.234	<.001	1.078	0.452	.017	1.141	0.462	.014	1.819	0.530	.001	1.854	0.594	.002
Satisfaction t_1	1.272	0.141	<.001	1.277	0.143	<.001	2.248	0.251	<.001	2.248	0.252	<.001	2.656	0.241	<.001	2.659	0.242	<.001
Satisfaction t_2	1.090	0.268	<.001	1.091	0.267	<.001	1.692	0.451	<.001	1.724	0.457	<.001	1.726	0.535	.001	1.716	0.559	.002
Intercept (t_0)	1.335	0.236	<.001	1.333	0.238	<.001	2.393	0.468	<.001	2.351	0.470	<.001	5.478	0.573	<.001	5.353	0.657	<.001
Intercept (t_2)	1.524	0.317	<.001	1.524	0.316	<.001	2.557	0.475	<.001	2.536	0.475	<.001	4.326	0.578	<.001	4.339	0.582	<.001
Linear slope	0.289	0.133	.030	0.282	0.134	.035	0.518	0.248	.037	0.482	0.255	.058	0.837	0.305	.006	0.813	0.349	.020

Note. *N* = 1,536. Regression coefficients and intercept–slope covariances significant at the $p < .05$ level are in bold type.
