# Supplementary material for: Aspiration–attainment gaps predict adolescents’ subjective well-being after transition to vocational education and training in Germany
Source: PLoS One. 2023 Jun 12;18(6):e0287064. doi: 10.1371/journal.pone.0287064 (PMC10259778; doi:10.1371/journal.pone.0287064)
Supplement: S8 Appendix — (PDF) [file pone.0287064.s008.pdf]

S8 Appendix

Unstandardized Coefficients of the Latent Growth Curve Models for Three Domains of Subjective Well-Being Regressed on the Aspiration–Attainment Gap and Covariates (Model III)

	General life satisfaction						Job satisfaction						Income satisfaction					
	Threshold 0			Threshold +/−5			Threshold 0			Threshold +/−5			Threshold 0			Threshold +/−5		
	Coef.	*SE*	*p*	Coef.	*SE*	*p*	Coef.	*SE*	*p*	Coef.	*SE*	*p*	Coef.	*SE*	*p*	Coef.	*SE*	*p*
Intercept (t_0) on																		
Underachievement	−0.188	0.135	.163	−0.203	0.136	.137	**−0.350**	0.176	.046	**−0.415**	0.183	.023	**−0.623**	0.250	.013	**−0.916**	0.252	<.001
Overachievement	**−0.436**	0.185	.018	−0.404	0.239	.091	**−0.651**	0.263	.013	−0.594	0.330	.072	−0.493	0.328	.133	**−0.917**	0.411	.026
Both groups combined[a]	***−0.270***	*0.124*	*.030*	*−0.256*	*0.130*	*.050*	***−0.452***	*0.168*	*.007*	***−0.461***	*0.178*	*.009*	***−0.576***	*0.232*	*.013*	***−0.918***	*0.238*	*<.001*
Intercept (t_2) on																		
Underachievement	−0.259	0.194	.181	−0.180	0.209	.389	−0.403	0.238	.090	−0.199	0.255	.435	−0.153	0.268	.567	−0.258	0.285	.366
Overachievement	−0.212	0.178	.235	−0.102	0.215	.634	−0.245	0.305	.422	0.098	0.322	.768	−0.170	0.360	.636	−0.188	0.421	.655
Intercept (t_0) on																		
Conscientiousness	**0.094**	0.041	.022	**0.100**	0.041	.014	0.095	0.061	.123	0.100	0.060	.094	0.109	0.091	.231	0.110	0.089	.218
Extraversion	**0.084**	0.042	.047	**0.085**	0.042	.045	0.113	0.058	.052	0.113	0.058	.052	−0.062	0.074	.403	−0.057	0.074	.443
Agreeableness	**0.218**	0.053	<.001	**0.215**	0.053	<.001	0.108	0.080	.176	0.104	0.079	.190	**0.275**	0.105	.009	**0.269**	0.105	.011
Openness	0.082	0.049	.094	0.080	0.049	.104	0.029	0.076	.700	0.033	0.075	.661	0.161	0.103	.118	0.162	0.102	.112
Emotional Stability	**0.164**	0.044	<.001	**0.169**	0.045	<.001	**0.166**	0.065	.011	**0.175**	0.066	.008	0.058	0.086	.503	0.074	0.087	.396
Parental SES	0.002	0.004	.603	0.002	0.004	.551	−0.004	0.007	.520	−0.004	0.007	.531	**0.019**	0.008	.018	**0.019**	0.008	.015
Migration background	0.247	0.126	.050	0.240	0.125	.055	0.042	0.192	.826	0.028	0.192	.884	0.118	0.258	.649	0.114	0.257	.656
Female	−0.082	0.095	.385	−0.070	0.095	.462	−0.170	0.140	.225	−0.158	0.140	.257	−0.148	0.191	.438	−0.077	0.192	.687
Post-economic crisis recovery	−0.125	0.111	.262	−0.128	0.110	.246	−0.190	0.158	.229	−0.210	0.157	.182	−0.033	0.206	.874	−0.065	0.205	.752
Pre-economic crisis	**−0.325**	0.119	.006	**−0.320**	0.117	.006	**−0.391**	0.166	.018	**−0.396**	0.168	.018	**−0.635**	0.245	.010	**−0.675**	0.245	.006
VET entry before 1st interview	**0.335**	0.105	.001	**0.342**	0.104	.001	−0.339	0.302	.262	−0.352	0.303	.246	0.360	0.339	.288	0.302	0.337	.371
Intercept (t_2) on																		
Conscientiousness	−0.004	0.055	.945	0.002	0.055	.969	0.113	0.086	.192	0.124	0.086	.151	**0.241**	0.096	.012	**0.240**	0.096	.012
Extraversion	0.073	0.058	.202	0.073	0.058	.207	0.045	0.087	.604	0.046	0.087	.597	−0.134	0.084	.113	−0.129	0.085	.129
Agreeableness	0.097	0.073	.189	0.096	0.073	.193	**0.244**	0.101	.016	**0.241**	0.102	.019	0.127	0.111	.253	0.124	0.111	.266
Openness	−0.018	0.067	.784	−0.025	0.067	.713	−0.017	0.095	.858	−0.030	0.094	.753	−0.046	0.102	.655	−0.047	0.102	.649
Emotional Stability	**0.306**	0.067	<.001	**0.306**	0.068	<.001	**0.194**	0.090	.031	**0.193**	0.089	.030	0.062	0.095	.511	0.064	0.094	.495

(continued)

	General life satisfaction						Job satisfaction						Income satisfaction					
	Threshold 0			Threshold +/−5			Threshold 0			Threshold +/−5			Threshold 0			Threshold +/−5		
	Coef.	*SE*	*p*	Coef.	*SE*	*p*	Coef.	*SE*	*p*	Coef.	*SE*	*p*	Coef.	*SE*	*p*	Coef.	*SE*	*p*
Intercept (t_2) on																		
Parental SES	−0.005	0.006	.370	−0.004	0.006	.466	**−0.029**	0.008	<.001	**−0.027**	0.008	.001	**0.023**	0.009	.009	**0.023**	0.009	.010
Migration background	0.130	0.172	.449	0.143	0.174	.411	−0.319	0.237	.179	−0.276	0.241	.253	0.235	0.301	.435	0.245	0.306	.424
Female	−0.035	0.135	.794	−0.030	0.136	.826	−0.264	0.185	.153	−0.265	0.185	.153	−0.085	0.215	.693	−0.063	0.216	.770
Post-crisis recovery	**−0.366**	0.171	.032	**−0.353**	0.170	.038	−0.230	0.210	.273	−0.196	0.208	.346	−0.395	0.243	.104	−0.411	0.242	.090
Pre-economic crisis	**−0.507**	0.166	.002	**−0.497**	0.165	.003	−0.351	0.252	.164	−0.341	0.251	.174	**−1.010**	0.274	<.001	**−0.996**	0.275	<.001
VET entry before 1st interview	−0.142	0.169	.401	−0.114	0.171	.506	−0.241	0.227	.288	−0.183	0.226	.418	0.168	0.252	.504	0.152	0.251	.545
Linear slope on																		
Underachievement	−0.035	0.114	.756	0.011	0.120	.926	−0.026	0.134	.846	0.108	0.141	.445	0.235	0.167	.160	0.329	0.176	.061
Overachievement	0.112	0.113	.320	0.151	0.134	.259	0.203	0.195	.299	0.346	0.224	.123	0.161	0.198	.416	0.365	0.222	.101
Both groups combined[a]	*0.013*	*0.096*	*.889*	*0.049*	*0.102*	*.629*	*0.052*	*0.127*	*.682*	*0.185*	*0.131*	*.157*	*0.207*	*0.150*	*.168*	***0.337***	*0.156*	*.031*
Linear slope on																		
Conscientiousness	−0.049	0.032	.129	−0.049	0.032	.128	0.009	0.052	.860	0.012	0.051	.820	0.066	0.059	.261	0.065	0.058	.262
Extraversion	−0.006	0.033	.867	−0.006	0.033	.856	−0.034	0.049	.483	−0.033	0.048	.491	−0.036	0.049	.460	−0.036	0.049	.458
Agreeableness	−0.061	0.043	.159	−0.060	0.043	.163	0.068	0.061	.264	0.068	0.061	.264	−0.074	0.067	.267	−0.073	0.067	.275
Openness	−0.050	0.040	.210	−0.052	0.040	.187	−0.023	0.059	.697	−0.031	0.058	.594	−0.103	0.065	.110	−0.104	0.064	.103
Emotional Stability	0.071	0.040	.075	0.068	0.040	.087	0.014	0.053	.789	0.009	0.052	.866	0.002	0.056	.969	−0.005	0.056	.934
Parental SES	−0.004	0.003	.295	−0.003	0.003	.340	**−0.012**	0.005	.015	**−0.011**	0.005	.024	0.002	0.005	.672	0.002	0.006	.699
Migration background	−0.058	0.106	.583	−0.048	0.107	.652	−0.181	0.147	.220	−0.152	0.149	.307	0.059	0.179	.744	0.065	0.181	.719
Female	0.024	0.079	.766	0.020	0.080	.801	−0.047	0.110	.669	−0.053	0.110	.627	0.032	0.129	.807	0.007	0.130	.955
Post-economic crisis recovery	−0.121	0.098	.216	−0.112	0.097	.248	−0.020	0.127	.875	0.007	0.127	.956	−0.181	0.145	.214	−0.173	0.145	.234
Pre-economic crisis	−0.091	0.100	.360	−0.088	0.099	.371	0.020	0.144	.890	0.027	0.145	.850	−0.188	0.159	.238	−0.160	0.158	.310
VET entry before 1st interview	**−0.238**	0.100	.017	**−0.228**	0.100	.023	0.049	0.210	.816	0.084	0.209	.688	−0.096	0.222	.667	−0.075	0.221	.733
Intercept–slope																		
Covariance (t_0)	−0.185	0.108	.088	−0.179	0.135	.185	**−0.590**	0.284	.038	−0.542	0.291	.062	**−1.067**	0.353	.002	**−1.001**	0.362	.006
Covariance (t_2)	0.284	0.166	.087	0.282	0.165	.088	0.433	0.288	.133	0.423	0.293	.149	0.498	0.339	.142	0.512	0.342	.135
Means																		
Underachievement	0.347	0.016	<.001	0.289	0.015	<.001	0.347	0.016	<.001	0.288	0.015	<.001	0.351	0.016	<.001	0.294	0.015	<.001
Overachievement	0.152	0.013	<.001	0.096	0.011	<.001	0.151	0.013	<.001	0.096	0.011	<.001	0.152	0.013	<.001	0.097	0.011	<.001

(continued)

	General life satisfaction						Job satisfaction						Income satisfaction					
	Threshold 0			Threshold +/−5			Threshold 0			Threshold +/−5			Threshold 0			Threshold +/−5		
	Coef.	*SE*	*p*	Coef.	*SE*	*p*	Coef.	*SE*	*p*	Coef.	*SE*	*p*	Coef.	*SE*	*p*	Coef.	*SE*	*p*
Means																		
Conscientiousness	4.951	0.028	<.001	4.951	0.028	<.001	4.951	0.028	<.001	4.951	0.028	<.001	4.951	0.028	<.001	4.951	0.028	<.001
Extraversion	4.900	0.032	<.001	4.900	0.032	<.001	4.900	0.032	<.001	4.901	0.032	<.001	4.900	0.032	<.001	4.900	0.032	<.001
Agreeableness	5.351	0.024	<.001	5.351	0.024	<.001	5.351	0.024	<.001	5.351	0.024	<.001	5.351	0.024	<.001	5.351	0.024	<.001
Openness	4.638	0.026	<.001	4.639	0.026	<.001	4.638	0.026	<.001	4.639	0.026	<.001	4.638	0.026	<.001	4.639	0.026	<.001
Emotional Stability	4.047	0.029	<.001	4.047	0.029	<.001	4.048	0.029	<.001	4.047	0.029	<.001	4.047	0.029	<.001	4.047	0.029	<.001
Parental SES	43.180	1.111	<.001	42.977	1.127	<.001	43.049	1.093	<.001	42.839	1.113	<.001	43.114	1.107	<.001	42.928	1.120	<.001
Migration background	0.189	0.010	<.001	0.189	0.010	<.001	0.189	0.010	<.001	0.189	0.010	<.001	0.189	0.010	<.001	0.189	0.010	<.001
Female	0.452	0.013	<.001	0.452	0.013	<.001	0.452	0.013	<.001	0.452	0.013	<.001	0.452	0.013	<.001	0.452	0.013	<.001
Post-economic crisis recovery	0.339	0.012	<.001	0.339	0.012	<.001	0.339	0.012	<.001	0.339	0.012	<.001	0.339	0.012	<.001	0.339	0.012	<.001
Pre-economic crisis	0.213	0.010	<.001	0.213	0.010	<.001	0.213	0.010	<.001	0.213	0.010	<.001	0.213	0.010	<.001	0.213	0.010	<.001
VET entry before 1st interview	0.233	0.011	<.001	0.233	0.011	<.001	0.233	0.011	<.001	0.233	0.011	<.001	0.233	0.011	<.001	0.233	0.011	<.001
Intercepts																		
Intercept (t_0)	4.689	0.457	<.001	4.610	0.453	<.001	6.193	0.632	<.001	6.091	0.627	<.001	3.080	0.828	<.001	3.066	0.816	<.001
Intercept (t_2)	6.001	0.620	<.001	5.890	0.615	<.001	6.202	0.764	<.001	5.984	0.754	<.001	3.866	0.887	<.001	3.872	0.881	<.001
Linear slope	0.656	0.376	.081	0.640	0.372	.086	0.004	0.497	.993	−0.054	0.490	.913	0.393	0.537	.465	0.403	0.533	.450
Variances																		
Underachievement	0.224	0.006	<.001	0.201	0.007	<.001	0.223	0.006	<.001	0.200	0.007	<.001	0.223	0.006	<.001	0.200	0.007	<.001
Overachievement	0.125	0.009	<.001	0.083	0.009	<.001	0.125	0.009	<.001	0.083	0.009	<.001	0.125	0.009	<.001	0.083	0.009	<.001
Conscientiousness	1.215	0.042	<.001	1.215	0.042	<.001	1.215	0.042	<.001	1.215	0.042	<.001	1.215	0.042	<.001	1.215	0.042	<.001
Extraversion	1.552	0.052	<.001	1.552	0.052	<.001	1.552	0.052	<.001	1.552	0.052	<.001	1.552	0.052	<.001	1.552	0.052	<.001
Agreeableness	0.856	0.032	<.001	0.856	0.032	<.001	0.856	0.032	<.001	0.856	0.032	<.001	0.856	0.032	<.001	0.856	0.032	<.001
Openness	0.993	0.033	<.001	0.993	0.033	<.001	0.993	0.033	<.001	0.993	0.033	<.001	0.993	0.033	<.001	0.993	0.033	<.001
Emotional Stability	1.309	0.044	<.001	1.309	0.044	<.001	1.309	0.044	<.001	1.309	0.044	<.001	1.309	0.044	<.001	1.309	0.044	<.001
Parental SES	347.583	14.889	<.001	347.121	14.505	<.001	348.672	14.728	<.001	347.736	14.365	<.001	346.969	14.804	<.001	346.421	14.453	<.001
Migration background	0.154	0.006	<.001	0.154	0.006	<.001	0.154	0.006	<.001	0.154	0.006	<.001	0.154	0.006	<.001	0.154	0.006	<.001
Female	0.248	0.001	<.001	0.248	0.001	<.001	0.248	0.001	<.001	0.248	0.001	<.001	0.248	0.001	<.001	0.248	0.001	<.001
Post-economic crisis recovery	0.224	0.004	<.001	0.224	0.004	<.001	0.224	0.004	<.001	0.224	0.004	<.001	0.224	0.004	<.001	0.224	0.004	<.001
Pre-economic crisis	0.168	0.006	<.001	0.168	0.006	<.001	0.168	0.006	<.001	0.168	0.006	<.001	0.168	0.006	<.001	0.168	0.006	<.001
VET entry before 1st interview	0.179	0.006	<.001	0.179	0.006	<.001	0.179	0.006	<.001	0.179	0.006	<.001	0.179	0.006	<.001	0.179	0.006	<.001

(continued)

	General life satisfaction						Job satisfaction						Income satisfaction					
	Threshold 0			Threshold +/−5			Threshold 0			Threshold +/−5			Threshold 0			Threshold +/−5		
	Coef.	*SE*	*p*	Coef.	*SE*	*p*	Coef.	*SE*	*p*	Coef.	*SE*	*p*	Coef.	*SE*	*p*	Coef.	*SE*	*p*
Residual variances																		
Satisfaction t_0	1.202	0.206	<.001	1.211	0.224	<.001	0.974	0.442	.028	1.037	0.452	.022	1.874	0.522	<.001	1.905	0.528	<.001
Satisfaction t_1	1.262	0.137	<.001	1.266	0.137	<.001	2.286	0.244	<.001	2.284	0.245	<.001	2.638	0.243	<.001	2.646	0.243	<.001
Satisfaction t_2	1.127	0.228	<.001	1.130	0.264	<.001	1.716	0.441	<.001	1.738	0.447	<.001	1.745	0.507	.001	1.737	0.509	.001
Intercept (t_0)	1.137	0.217	<.001	1.133	0.222	<.001	2.363	0.451	<.001	2.312	0.453	<.001	5.079	0.558	<.001	4.939	0.568	<.001
Intercept (t_2)	1.336	0.298	<.001	1.339	0.297	<.001	2.047	0.472	<.001	2.074	0.473	<.001	3.942	0.554	<.001	3.960	0.560	<.001
Linear slope	0.234	0.069	.001	0.230	0.123	.060	0.511	0.239	.032	0.482	0.246	.050	0.783	0.290	.007	0.756	0.296	.011

Note. VET = vocational education and training. N = 1,536. Regression coefficients and intercept–slope covariances significant at the $p < .05$ level are in bold type.

[a] For further analyses, which we briefly address only in the Discussion section of our paper, we combined under- and overachievement in one group.
